# Variation in 5-hydroxymethylcytosine across human cortex and cerebellum

**DOI:** 10.1186/s13059-016-0871-x

**Published:** 2016-02-16

**Authors:** Katie Lunnon, Eilis Hannon, Rebecca G. Smith, Emma Dempster, Chloe Wong, Joe Burrage, Claire Troakes, Safa Al-Sarraj, Agnieszka Kepa, Leonard Schalkwyk, Jonathan Mill

**Affiliations:** University of Exeter Medical School, RILD, University of Exeter, Barrack Road, Devon, UK; Institute of Psychiatry, Psychology and Neuroscience, King’s College London, De Crespigny Park, London, UK; University of Essex, Wivenhoe Park, Colchester, CO4 3SQ UK

**Keywords:** Epigenetics, DNA methylation, Brain, 5-methylcytosine, 5mC, 5-hydroxymethylcytosine, 5hmC, EWAS, Illumina Infinium 450K Beadarray, Cerebellum

## Abstract

**Background:**

The most widely utilized approaches for quantifying DNA methylation involve the treatment of genomic DNA with sodium bisulfite; however, this method cannot distinguish between 5-methylcytosine (5mC) and 5-hydroxymethylcytosine (5hmC). Previous studies have shown that 5hmC is enriched in the brain, although little is known about its genomic distribution and how it differs between anatomical regions and individuals. In this study, we combine oxidative bisulfite (oxBS) treatment with the Illumina Infinium 450K BeadArray to quantify genome-wide patterns of 5hmC in two distinct anatomical regions of the brain from multiple individuals.

**Results:**

We identify 37,145 and 65,563 sites passing our threshold for detectable 5hmC in the prefrontal cortex and cerebellum respectively, with 23,445 loci common across both brain regions. Distinct patterns of 5hmC are identified in each brain region, with notable differences in the genomic location of the most hydroxymethylated loci between these brain regions. Tissue-specific patterns of 5hmC are subsequently confirmed in an independent set of prefrontal cortex and cerebellum samples.

**Conclusions:**

This study represents the first systematic analysis of 5hmC in the human brain, identifying tissue-specific hydroxymethylated positions and genomic regions characterized by inter-individual variation in DNA hydroxymethylation. This study demonstrates the utility of combining oxBS-treatment with the Illumina 450k methylation array to systematically quantify 5hmC across the genome and the potential utility of this approach for epigenomic studies of brain disorders.

**Electronic supplementary material:**

The online version of this article (doi:10.1186/s13059-016-0871-x) contains supplementary material, which is available to authorized users.

## Background

Epigenetic modifications to DNA play a critical role in establishing and maintaining cellular phenotype [1]. Recent studies highlight widespread changes in DNA methylation occurring during neurodevelopment, with tissue-specific methylomic variation present between discrete regions of the human brain [2, 3]. Epigenetic processes control key neurobiological and cognitive processes in the brain, and their importance is highlighted by evidence implicating methylomic variation in a number of neuropsychiatric and neurodegenerative diseases, including multiple sclerosis, autism, Alzheimer’s disease, and schizophrenia [4–7].

Although 5-methylcytosine (5mC) is the best understood and most studied epigenetic modification modulating transcriptional plasticity in the mammalian genome, three additional DNA modifications (5-hydroxymethylcytosine (5hmC), 5-formylcytosine (5fC), and 5-carboxylcytosine (5caC)) have been recently described. These modifications are thought to represent intermediates in the demethylation of 5mC to un-modified cytosine [8] although recent data suggest there are specific functional roles for 5hmC. For example, 5hmC is specifically recognized by key binding-proteins [9], and can be maintained through cell division [10]. The exact genomic distribution of 5hmC is still debated; some studies have reported 5hmC in gene promoters and gene bodies [11, 12], while others have shown a depletion of 5hmC in CpG islands and an enrichment outside of CG-rich regions [13, 14]. It has been shown that 5hmC occurs at relatively high levels in the cerebellum and other regions of the brain [15, 16], where it is particularly enriched in the vicinity of genes with synapse-related functions [17]. Of note, recent studies have reported global alterations in 5hmC in Alzheimer’s disease [18, 19], supporting a role in health and disease.

Until recently it has not been possible to sensitively quantify 5hmC at base-pair resolution in the genome across large numbers of samples. Furthermore, many of the existing methods routinely used to interrogate DNA methylation (that is, those based on sodium bisulfite (BS) conversion and methylation-sensitive restriction enzyme cleavage) are unable to discriminate between 5mC and 5hmC [20]. The recent development of oxidative bisulfite (oxBS) treatment [13, 21], however, which involves the oxidation of 5hmC to 5fC before BS conversion, allows both a direct measurement of absolute 5mC and a proxy measure of 5hmC. Two recent papers demonstrated that oxBS conversion can be integrated with the Illumina 450K HumanMethylation (450K) array to facilitate the systematic quantification of both 5mC and 5hmC across the genome [22, 23]. In this study we used a commercially available oxBS treatment kit (TrueMethyl-CEGX, Cambridge, UK) in conjunction with the Illumina 450K array to compare the distribution of 5hmC across two regions of the human brain (prefrontal cortex and cerebellum) dissected from eight donors. We subsequently confirmed our findings in an independent set of matched prefrontal cortex and cerebellum samples dissected from an additional 18 individuals.

## Results and discussion

### Identifying differences in hydroxymethylated sites between cortex and cerebellum

The aim of the study was to compare 5hmC in matched postmortem prefrontal cortex and cerebellum samples from multiple donors using a commercially available oxBS conversion kit in combination with the Illumina 450K Human Methylation array. Briefly, the level of 5hmC at specific sites is quantified by subtracting oxBS-generated 450K array profiles from those generated following a BS-conversion performed in parallel. Each sample in this study was also profiled following a standard BS-conversion protocol using the Zymo EZ DNA methylation kit. Following normalization, the distribution of beta values was highly consistent across both BS methods (CEGX vs. Zymo) (Additional file 1: Figure S1A), with a highly significant correlation observed in both the prefrontal cortex (Additional file 1: Figure S1B; R^2^ = 0.99, *P* <2.2E-16) and cerebellum samples (Additional file 1: Figure S1C; R^2^ = 0.99, *P <*2.2E-16). These data indicate that the CEGX BS conversion protocol yields data that are directly comparable to data generated using standard BS conversion kits widely employed prior to 450K array processing.

**Fig. 1:**
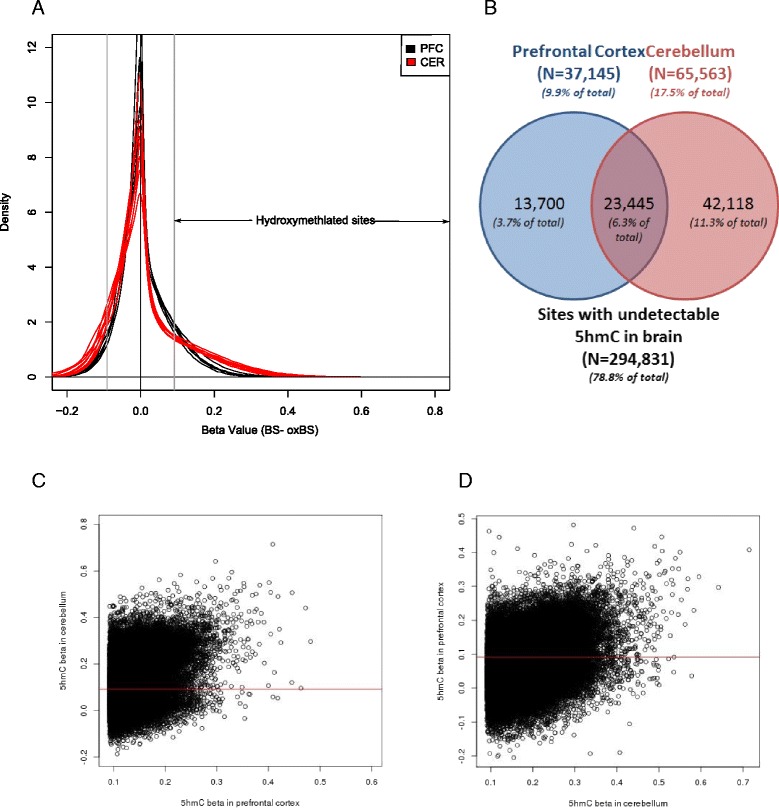
Quantifying 5hmC in two regions of the human brain. **a** Δβ_BS-oxBS_ was calculated for each sample and a detection threshold based on the lowest fifth percentile in the negative values (0.09158275) used to call ‘detectable’ 5hmC (black vertical line). **b** We identified 37,145 and 65,563 probes with a mean 5hmC level above threshold in the prefrontal cortex and cerebellum, respectively. **c** The degree of hydroxymethylation at sites with ‘detectable’ 5hmC in prefrontal cortex (N = 37,145) is correlated with levels at the same sites in the cerebellum (adjusted R2 = 0.097, *P* <2.2E-16). The red horizontal line indicates our threshold for ‘detectable’ 5hmC. **d** The degree of hydroxymethylation at sites with ‘detectable’ 5hmC in cerebellum (N = 65,563) is correlated with levels at the same sites in the prefrontal cortex (adjusted R2 = 0.132, *P* <2.2E-16)

We were interested in establishing the location of sites characterized by ‘detectable’ 5hmC and, building on our previous data demonstrating region-specific patterns of 5mC in the human brain [2], the extent to which levels of 5hmC differed between the prefrontal cortex and cerebellum. 5hmC levels were calculated by subtracting the oxBS beta-value from the BS beta value at each probe on the 450K array (Δβ_BS-oxBS_) (see Methods). As expected, the distribution of Δβ_BS-oxBS_ values was positively-skewed (Fig. 1a), although a small proportion of probes in each sample were characterized by a negative Δβ_BS-oxBS_ value, likely resulting from technical variance inherent in the Illumina array protocol. We therefore set a stringent threshold for calling 5hmC based on the 95th percentile of negative Δβ_BS-oxBS_ values across all profiled samples, to ensure we only analyzed probes characterized by ‘detectable’ levels of 5hmC. In this dataset, therefore, only sites with an average Δβ_BS-oxBS_ level in either tissue >0.09158275 were classified as having ‘detectable’ levels of 5hmC. Using this threshold, we identified a total of 79,263 loci characterized by ‘detectable’ 5hmC in one or both brain regions.

Of note, there was a striking difference in the prevalence of 5hmC-positive sites between the prefrontal cortex and cerebellum (Fig. 1a); we identified 37,145 (13,700 unique) and 65,563 (42,118 unique) probes with an average 5hmC level above threshold in the prefrontal cortex and cerebellum, respectively, with 23,445 probes characterized by ‘detectable’ 5hmC in both regions of the brain (Additional file 2: Table S1, Additional file 2: Table S2, and Fig. 1b). Of the 37,145 sites with ‘detectable’ 5hmC in the prefrontal cortex we observed a small but significant correlation with 5hmC level at the same sites in the cerebellum (Fig. 1c; adjusted R^2^ = 0.097, *P* <2.2E-16). Similarly for the 65,563 sites with ‘detectable’ 5hmC in the cerebellum we observed a significant correlation with 5hmC in the prefrontal cortex (Fig. 1d; adjusted R^2^ = 0.132, *P* <2.2E-16). As a resource to other researchers interested in the distribution of 5hmC in the brain, average Δβ_BS-oxBS_ levels for each of the 79,263 probes on the 450K array characterized by ‘detectable’ 5hmC in one or both brain regions can be explored in the Hydroxymethylation Annotation in Brain Integrative Tool (HABIT) at our laboratory website (http://epigenetics.iop.kcl.ac.uk/HMC/). The tool also integrates annotated UCSC tracks to enable visualization of average 5hmC levels in both brain regions.

### The distribution of 5hmC differs depending on genic location and CG density

**Fig. 2:**
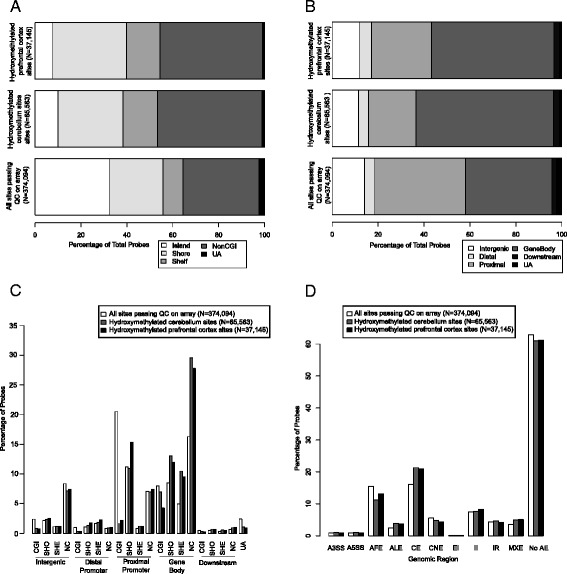
Hydroxymethylation is enriched in specific genomic features in brain. **a** Hydroxymethylated loci are significantly under-enriched in CpG islands, shores, and shelves and (**b**, **c**) significantly enriched in the gene body. **d** Across both brain regions sites with ‘detectable’ 5hmC are enriched in alternative last exons, cassette exons, and mutually exclusive exons, and under-enriched in alternative first exons and constitutive exons. The level of enrichment was determined by Fisher’s exact test. QC: quality control; CGI: CpG island; SHO: CpG island shore; SHE: CpG island shelf; nonCGI, NC: outside CpG islands; UA: unannotated; A3SS: 3’ splice site; A5SS: alternative 5’ splice site; AFE: alternative first exon; ALE: alternative last exon; CE: cassette exon; CNE: constitutive exon; EI: exon isoforms; II: intron isoforms; IR: intron retention; MXE: mutually exclusive exon

Given that the abundance of 5mC is known to vary across the genome, we were interested in whether there is an enrichment of 5hmC in certain annotated regions of the genome. Although the Illumina 450K array does not enable an assessment of all potentially hydroxymethylated probes in the human genome, it is the most widely-used tool in epigenetic epidemiology and covers 99 % of RefSeq genes, with an average of 17 CpG sites per gene region distributed across the promoter, 5'UTR, first exon, gene body, and 3'UTR. We found that ‘detectable’ 5hmC is highly depleted in CpG islands in both brain regions (prefrontal cortex: OR = 0.18, *P* <2.53E-294; cerebellum: OR = 0.23, *P* <2.53E-294). In contrast, 5hmC is enriched in CpG island shores (prefrontal cortex: OR = 1.55, *P* = 2.53E-294; cerebellum: OR = 1.30, *P* = 1.80E-159), CpG island shelves (prefrontal cortex: OR = 1.78, *P* = 3.93E-262; cerebellum: OR = 1.86, *P* <2.53E-294), and locations outside of CG-rich regions (prefrontal cortex: OR = 1.62, *P* <2.53E-294; cerebellum: OR = 1.68, *P* <2.53E-294) (Table 1, Fig. 2a). This is consistent with previous studies demonstrating a depletion of 5hmC in CpG islands and an enrichment outside of CG-rich regions [13, 14]. Furthermore, ‘detectable’ 5hmC was significantly enriched in both brain regions in the gene body (prefrontal cortex: OR = 1.90, *P* <2.53E-294; cerebellum: OR = 2.48, *P* <2.53E-294), (Table 1, Fig. 2b, c), and also downstream of annotated transcripts (prefrontal cortex: OR = 1.30, *P* = 1.95E-12; cerebellum: OR = 1.35, *P* = 2.04E-25). In contrast, in both brain regions, 5hmC was depleted at intergenic sites (prefrontal cortex: OR = 0.82, *P* = 8.46E-34; cerebellum: OR = 0.79, *P* = 5.13E-75) and the proximal promoter (prefrontal cortex: OR = 0.54, *P* <2.53E-294; cerebellum: OR = 0.40, *P* <2.53E-294). This is consistent with previous studies showing a decrease in brain 5hmC in intergenic regions [24] and an enrichment of 5hmC in gene bodies [22]. Interestingly, 5hmC was modestly enriched in distal promoter sites in the prefrontal cortex (OR = 1.19, *P* = 5.16E-12), but not in the cerebellum (OR = 0.97, *P* = 0.166). These data concur with previous studies using oxBS in conjunction with the 450K array in smaller numbers of samples. Stewart *et al*. demonstrated a significant enrichment of probes with detectable 5hmC in gene bodies when investigating one unmatched cerebellum and frontal cortex sample [22], while Field *et al*. showed that loci with detectable 5hmC are enriched in the gene body (exonic and intronic) and regions downstream of the gene in a single cerebellum sample [23].

**Table 1:** Specific genomic features are characterized by hydroxymethylation in brain

Probes passing QC on array (%)		Prefrontal cortex			Cerebellum		
		Detectable 5hmC sites (%)	Enrichment (95 % CI)	*P* value	Detectable 5hmC sites (%)	Enrichment (95 % CI)	*P* value
All probes (N = 374,094)		37,145	-	-	65,563	-	-
CpG island feature							
Island	120,922 (32.3 %)	2,868 (7.7 %)	0.18 (0.17–0.18)	<2.53E-294	6,524 (10.0 %)	0.23 (0.23–0.24)	<2.53E-294
Shore	87,312 (23.3 %)	11,934 (32.1 %)	1.55 (1.52–1.59)	2.53E-294	18,544 (28.3 %)	1.30 (1.27–1.32)	1.80E-159
Shelf	32,912 (8.8 %)	5,434 (14.6 %)	1.78 (1.72–1.83)	3.93E-262	9,958 (15.2 %)	1.86 (1.81–1.90)	<2.53E-294
Outside	124,010 (33.1 %)	16,565 (44.6 %)	1.62 (1.59–1.66)	<2.53E-294	29,831 (45.5 %)	1.68 (1.66–1.71)	<2.53E-294
Unannotated	8,938 (2.4 %)	344 (0.9 %)	0.38 (0.34–0.43)	2.99E-91	706 (1.1 %)	0.44 (0.41–0.48)	9.15E-118
Gene feature							
Intergenic	52,325 (14 %)	4,367 (11.8 %)	0.82 (0.79–0.85)	8.46E-34	7,461 (11.4 %)	0.79 (0.77–0.81)	5.13E-75
Distal promoter	16,990 (4.5 %)	1,986 (5.3 %)	1.19 (1.13–1.25)	5.16E-12	2,897 (4.4 %)	0.97 (0.93–1.01)	0.166
Proximal promoter	148,029 (39.6 %)	9,693 (26.1 %)	0.54 (0.53–0.55)	<2.53E-294	13,544 (20.7 %)	0.40 (0.39–0.41)	<2.53E-294
Gene body	140,919 (37.7 %)	19,870 (53.5 %)	1.90 (1.86–1.94)	<2.53E-294	39,335 (60.0 %)	2.48 (2.44–2.52)	<2.53E-294
Downstream	6,893 (1.8 %)	885 (2.4 %)	1.30 (1.21–1.40)	1.95E-12	1,620 (2.5 %)	1.35 (1.28–1.43)	2.04E-25
Unannotated	8,938 (2.4 %)	344 (0.9 %)	0.38 (0.34–0.43)	2.99E-91	706 (1.1 %)	0.44 (0.41–0.48)	9.15E-118
Transcription factor binding site	184,178 (49.2 %)	14,623 (39.4 %)	0.67 (0.66–0.68)	8.40E-291	19,499 (29.7 %)	0.44 (0.43–0.44)	<2.53E-294
Dnase 1 hypersensitivity site	49,903 (13.3 %)	4,449 (12.0 %)	0.88 (0.86–0.91)	7.49E-14	6,227 (9.5 %)	0.68 (0.66–0.70)	5.80E-174
Alternative transcription events (N = 138,640 probes)		14,429	-	-	25,582	-	-
A3SS	3,347 (2.4 %)	330 (2.3 %)	0.95 (0.84–1.06)	0.361	700 (2.7 %)	1.14 (1.05–1.24)	2.63E-03
A5SS	3,364 (2.4 %)	357 (2.5 %)	1.02 (0.91–1.14)	0.712	710 (2.8 %)	1.15 (1.06–1.25)	1.11E-03
AFE	57,956 (41.8 %)	4,897 (33.9 %)	0.72 (0.69–0.74)	5.07E-76	7,332 (28.7 %)	0.56 (0.54–0.58)	<2.53E-294
ALE	9,026 (6.5 %)	1,353 (9.4 %)	1.49 (1.40–1.58)	1.55E-35	2,554 (10.0 %)	1.59 (1.52–1.67)	5.61E-81
CE	60,203 (43.4 %)	7,783 (53.9 %)	1.53 (1.47–1.58)	2.14E-128	13,980 (54.6 %)	1.57 (1.53–1.61)	1.17E-239
CNE	20,661 (14.9 %)	1,592 (11.0 %)	0.71 (0.67–0.75)	1.99E-38	3,149 (12.3 %)	0.80 (0.77–0.83)	3.28E-28
EI	137 (0.1 %)	19 (0.1 %)	1.33 (0.78–2.16)	0.270	45 (0.2 %)	1.78 (1.24–2.51)	1.39E-03
II	27,746 (20 %)	3,080 (21.3 %)	1.08 (1.04–1.13)	1.60E-04	4,974 (19.4 %)	0.96 (0.93–1.00)	0.036
IR	16,175 (11.7 %)	1,550 (10.7 %)	0.91 (0.86–0.96)	8.90E-04	3,025 (11.8 %)	1.02 (0.97–1.06)	0.472
MXE	13,054 (9.4 %)	1,880 (13 %)	1.44 (1.37–1.52)	8.63E-41	3,221 (12.6 %)	1.39 (1.33–1.44)	6.38E-52

The level of enrichment was determined by Fisher’s exact test. QC, quality control; 5hmC, 5-hydroxymethylcytosine; CI, confidence interval; A3SS, alternative 3’ splice site; A5SS, alternative 5’ splice site; AFE, alternative first exon; ALE, alternative last exon; CE, cassette exon; CNE, constitutive exon; EI, exon isoforms; II, intron isoforms; IR, intron retention; MXE, mutually exclusive exon

### 5hmC is enriched in certain functional elements and depleted in others

We used functional genomic annotation data from ENCODE [25, 26] to examine the distribution of 5hmC across regulatory regions of the genome in the brain. ‘Detectable’ 5hmC was found to be significantly depleted at transcription factor binding sites (TFBS) (prefrontal cortex: OR = 0.67, *P* = 8.40E-291; cerebellum: OR = 0.44, *P* <2.53E-294) and at DNAse I hypersensitivity sites (prefrontal cortex: OR = 0.88, *P* <7.49E-14; cerebellum: OR = 0.68, *P* = 5.80E-174) (Table 1).

We also examined alternative transcription events (Table 1; Fig. 2d) and found that 5hmC is significantly depleted in both brain regions at alternative first exons (AFE) (prefrontal cortex: OR = 0.72, *P* = 5.07E-76; cerebellum: OR = 0.56, *P* <2.53E-294) and constitutive exons (CNE) (prefrontal cortex: OR = 0.71, *P* = 1.99E-38; cerebellum: OR = 0.80, *P* = 3.28E-28). In contrast, 5hmC is significantly enriched in alternative last exons (ALE) (prefrontal cortex: OR = 1.49, *P* = 1.55E-35; cerebellum: OR = 1.59, *P* = 5.61E-81), cassette exons (CE) (prefrontal cortex: OR = 1.53, *P* = 2.14E-128; cerebellum: OR = 1.57, *P* = 1.17E-239), and mutually exclusive exons (MXE) (prefrontal cortex: OR = 1.44, *P* = 8.63E-41; cerebellum: OR = 1.39, *P* = 6.38E-52). This concurs with previous studies demonstrating elevated levels of 5hmC in CE in the human brain [17]. Furthermore, there is evidence of cerebellum-specific enrichment of 5hmC at alternative 3’ splice site (A3SS) (OR = 1.14, *P* = 2.63E-03), alternative 5’ splice site (A5SS) (OR = 1.15, *P* = 1.11E-03), and exon isoforms (EI) (OR = 1.78, *P* = 1.39E-03), with no equivalent enrichment in matched prefrontal cortex samples. Finally, there is depletion of 5hmC at intron retention (IR) events in the prefrontal cortex (OR = 0.91, *P* = 8.9E-04), but not the cerebellum (OR = 1.02, *P* = 0.472).

Using a logistic regression method to identify biological pathways enriched for loci annotated to sites with ‘detectable’ 5hmC, stringently controlling for the number probes annotated to each gene, we found considerable overlap in 5hmC-enriched pathways between the prefrontal cortex (Additional file 2: Table S3) and cerebellum (Additional file 2: Table S4), with the most significantly enriched pathway in both brain regions being nervous system development (prefrontal cortex: *P* = 1.5E-11; cerebellum: *P* = 4.1E-11).

### Levels of 5hmC at specific sites differ between prefrontal cortex and cerebellum

**Fig. 3:**
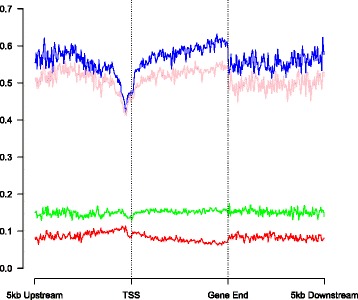
Gene-level analysis of canonical 5hmC. Levels of 5hmC are consistently higher in the cerebellum than in the prefrontal cortex along the length of the gene. Key: average 5hmC level in prefrontal cortex (red), average 5hmC level in cerebellum (green), average 5mC level in prefrontal cortex (blue), average 5mC level in cerebellum (pink)

After describing the genomic distribution of ‘detectable’ 5hmC, we were interested in estimating absolute levels of 5hmC at specific sites, and the extent to which these differ between brain regions and individuals. The canonical patterns of 5hmC and 5mC levels across the gene are shown in Fig. 3 for the 79,263 loci with detectable levels of 5hmC. 5mC levels across the gene are similar to those reported previously [2, 23], with a decrease in levels at the TSS, before a gradual increase through the gene body, and an eventual decrement downstream of the gene body. Interestingly although 5mC levels are similar at the TSS in both the prefrontal cortex and cerebellum, levels of 5mC were slightly elevated in the cerebellum at other regions along the gene, and more notably so at the 3’ end of the transcript. In contrast, 5hmC is characterized by a different genic pattern, with levels being consistently higher in the cerebellum than the prefrontal cortex across the entire gene, in addition to immediate upstream/downstream regions.

Additional file 2: Table S5 and Additional file 2: Table S6 list the 1,000 450K array sites with the highest estimated levels of 5hmC in the prefrontal cortex and cerebellum, respectively. Of the sites showing highest 5hmC in the prefrontal cortex (Additional file 2: Table S5), 349 did not exceed our detection threshold in the cerebellum. Similarly, of the sites showing highest 5hmC in the cerebellum (Additional file 2: Table S6), 651 did not exceed our detection threshold in the prefrontal cortex. These data suggest that although there is some similarity between brain regions, levels of 5hmC at individual sites are often tissue-specific.

**Fig. 4:**
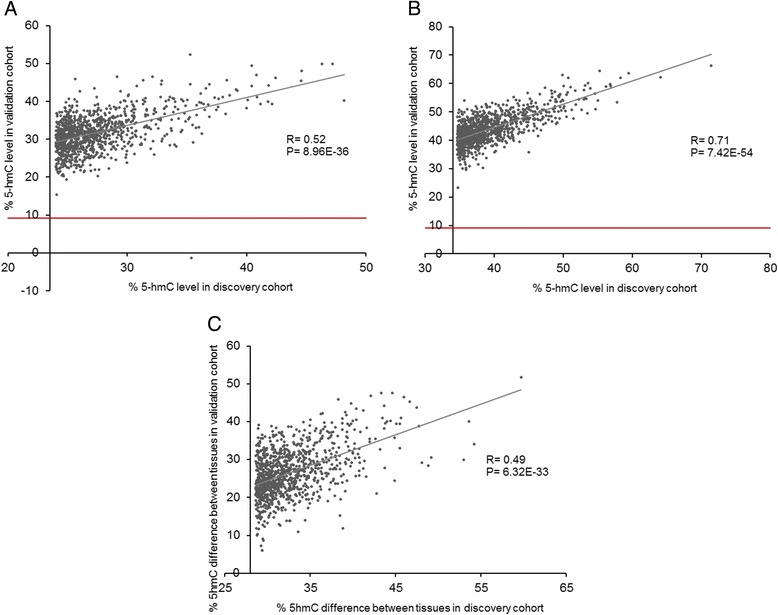
5hmC estimates generated using this approach were validated in an independent set of matched prefrontal cortex and cerebellum samples. Estimates of 5hmC at the 1,000 loci showing the highest levels of 5hmC in the discovery cohort were highly consistent and significantly correlated across datasets in both (**a**) the prefrontal cortex (R = 0.52, *P* = 8.96E-36) and (**b**) the cerebellum (R = 0.71, *P* = 7.42E-54). The red line denotes our threshold for robust 5hmC detection. (**c**) 5hmC differences between prefrontal cortex and cerebellum at the 1,000 top-ranked TS-HMPs identified in the discovery cohort were significantly correlated with differences identified at the same sites in the validation cohort (R = 0.49, *P* = 6.32E-33)

In order to confirm our findings, we subsequently examined 5hmC levels at the top 1,000 sites in additional matched prefrontal cortex and cerebellum samples dissected from 18 independent donors (Additional file 2: Tables S5; Additional file 2: Table S6). Estimates of 5hmC at these sites was highly concordant across datasets (median difference between discovery and replication datasets = 4.73 (prefrontal cortex) and 4.62 (cerebellum)). There is a highly significant correlation in estimated 5hmC levels between the discovery and validation datasets at these sites in both the prefrontal cortex (R = 0.52, *P* = 8.96E-36) and cerebellum (R = 0.71, *P* = 7.42E-54) (Fig. 4).

Additional file 2: Table S7 lists the top 1,000 sites characterized by the largest average differences in 5hmC between the prefrontal cortex and cerebellum, which we define as tissue-specific hydroxymethylated positions (TS-HMPs). Each of these 1,000 probes is characterized by at least 28 % hydroxymethylation difference between the brain regions. For the majority (96.5 %) of these probes, the difference was driven by increased 5hmC in the cerebellum compared to the prefrontal cortex (Additional file 1: Figure S2), although 35 of the 1,000 sites (3.5 %) did show elevated 5hmC in the prefrontal cortex compared to the cerebellum. A total of 997 out of the 1,000 loci showed a significant difference in 5hmC between the prefrontal cortex and cerebellum in our validation dataset (Additional file 2: Table S7), and there was a highly significant correlation in prefrontal cortex versus cerebellum differences at these loci between the two independent datasets (Fig. 4c; R = 0.49, *P* = 6.32E-33). Pathway analysis of these top 1,000 TS-HMPs showed an enrichment for various neurobiological processes that distinguish the cortex and cerebellum (Additional file 2: Table S8), for example acetylcholine binding (*P* = 1.51E-04), dopaminergic neuron differentiation (*P* = 1.03E-03), and cerebellar purkinje cell layer morphogenesis (*P* = 1.83E-03).

Although there is an overall depletion of ‘detectable’ 5hmC in CpG islands (Table 1) when compared to the genic distribution of all probes on the 450K array, it is notable that sites with the highest levels of 5hmC in both the prefrontal cortex and cerebellum are enriched in CpG islands (OR = 1.25, *P* = 0.022 and OR = 3.46, *P* = 7.01E-55, respectively) when compared to the distribution of the 79,263 sites with ‘detectable’ 5hmC (Table 2; Additional file 1: Figure S3A). Furthermore, we found a significant enrichment of TS-HMRs in CpG islands (OR = 4.23, *P* = 6.37E-79) (Table 3; Additional file 1: Figure S3A) when compared to the 79,263 sites with ‘detectable’ 5hmC. Conversely, TS-HMRs are depleted in both CpG island shores (OR = 0.66, *P* = 6.2E-08) and CpG island shelves (OR = 0.59, *P* = 2.64E-07). Interestingly we have previously shown that tissue-specific differentially methylated regions in the genome (TS-DMRs) are enriched in CpG island shores and shelves [2], indicating a clear distinction between the location of tissue-specific DNA methylation and hydroxymethylation. The most hydroxymethylated sites in the prefrontal cortex showed similar patterns with respect to an enrichment at TFBS (prefrontal cortex: OR = 2.53, *P* = 2.57E-47; cerebellum: OR = 1.31, *P* = 4.97E-05) and DHS (prefrontal cortex: OR = 1.54, *P* = 3.95E-06; cerebellum: OR = 1.45, *P* = 8.42E-05), but some tissue differences in their presence at alternative events (Additional file 1: Figure S3C), and within gene features (Additional file 1: Figure S3B). For example, the most hydroxymethylated sites are over-represented in the proximal promoter in the prefrontal cortex (OR = 2.11, *P* = 3.10E-27), but not the cerebellum, and there is an under-representation of the top hydroxymethylated sites at gene bodies in the prefrontal cortex (OR = 0.64, *P* = 5.30E-12), but an over-representation in the cerebellum (OR = 1.45, *P* = 2.60E-08). The previous study investigating 5hmC in a single cerebellum sample also demonstrated that the highest levels of 5hmC are found in the gene body, within introns [23].

**Table 2:** The most hydroxymethylated loci in prefrontal cortex and cerebellum are enriched in distinict genomic regions

Loci with detectable 5hmC in brain (%)		1,000 loci with highest 5hmC in prefrontal cortex			1,000 loci with highest 5hmC in cerebellum		
		Detectable 5hmC sites (%)	Enrichment (95 % CI)	*P* value	Detectable 5hmC sites (%)	Enrichment (95 % CI)	*P* value
All probes (N = 79,263)		1,000	-	-	1,000	-	-
CpG island feature							
Island	7,837 (9.9 %)	121 (12.1 %)	1.25 (1.03–1.52)	0.022	275 (27.5 %)	3.46 (2.99–3.99)	7.01E-55
Shore	22,593 (28.5 %)	472 (47.2 %)	2.24 (1.97–2.55)	3.19E-35	276 (27.6 %)	0.96 (0.83–1.10)	0.549
Shelf	11,674 (14.7 %)	93 (9.3 %)	0.59 (0.47–0.74)	4.29E-07	121 (12.1 %)	0.80 (0.65–0.97)	0.019
Outside	36,342 (45.8 %)	307 (30.7 %)	0.52 (0.46–0.60)	3.37E-22	326 (32.6 %)	0.57 (0.50–0.65)	2.94E-17
Unannotated	817 (1.0 %)	7 (0.7 %)	0.68 (0.27–1.41)	0.426	2 (0.2 %)	0.19 (0.02–0.70)	3.94E-03
Gene feature							
Intergenic	9,604 (12.1 %)	89 (8.9 %)	0.71 (0.56–0.88)	1.48E-03	74 (7.4 %)	0.58 (0.45–0.74)	1.56E-06
Distal promoter	3,703 (4.7 %)	46 (4.6 %)	0.98 (0.71–1.33)	1.000	19 (1.9 %)	0.40 (0.24–0.62)	4.88E-06
Proximal promoter	16,979 (21.4 %)	365 (36.5 %)	2.11 (1.85–2.40)	3.10E-27	206 (20.6 %)	0.95 (0.81–1.11)	0.561
Gene body	46,238 (58.3 %)	474 (47.4 %)	0.64 (0.57–0.73)	5.30E-12	670 (67.0 %)	1.45 (1.27–1.66)	2.60E-08
Downstream	1,922 (2.4 %)	19 (1.9 %)	0.78 (0.47–1.23)	0.350	29 (2.9 %)	1.20 (0.80–1.74)	0.351
Unannotated	817 (1.0 %)	7 (0.7 %)	0.68 (0.27–1.41)	0.426	2 (0.2 %)	0.19 (0.02–0.70)	3.94E-03
Transcription factor binding Site	25,482 (32.1 %)	545 (54.5 %)	2.53 (2.22–2.87)	2.57E-47	383 (38.3 %)	1.31 (1.15–1.49)	4.97E-05
Dnase 1 hypersensitivity site	8,155 (10.3 %)	150 (15 %)	1.54 (1.28–1.84)	3.95E-06	143 (14.3 %)	1.45 (1.21–1.74)	8.42E-05
Alternative transcription events (N = 30,659 probes)		398	-	-	451	-	-
A3SS	798 (2.6 %)	8 (2.0 %)	0.77 (0.33–1.54)	0.632	22 (4.9 %)	1.92 (1.18–2.96)	6.74E-03
A5SS	827 (2.7 %)	12 (3.0 %)	1.12 (0.57–1.90)	0.640	17 (3.8 %)	1.41 (0.81–2.30)	0.185
AFE	9,159 (29.9 %)	158 (39.7 %)	1.55 (1.25–1.90)	3.41E-05	112 (24.8 %)	0.78 (0.62–0.96)	0.020
ALE	3,017 (9.8 %)	27 (6.8 %)	0.67 (0.43–0.99)	0.041	46 (10.2 %)	1.04 (0.75–1.42)	0.811
CE	16,604 (54.2 %)	185 (46.5 %)	0.74 (0.60–0.90)	2.37E-03	208 (46.1 %)	0.72 (0.60–0.88)	7.17E-04
CNE	3,755 (12.2 %)	53 (13.3 %)	1.10 (0.81–1.48)	0.490	62 (13.7 %)	1.14 (0.86–1.50)	0.347
EI	50 (0.2 %)	1 (0.3 %)	1.54 (0.04–9.04)	0.482	2 (0.4 %)	2.73 (0.32–10.43)	0.174
II	6,003 (19.6 %)	89 (22.4 %)	1.18 (0.92–1.50)	0.163	87 (12.3 %)	0.98 (0.77–1.25)	0.952
IR	3,529 (11.5 %)	63 (15.8 %)	1.45 (1.08–1.90)	0.011	80 (17.7 %)	1.66 (1.28–2.12)	1.08E-04
MXE	3,814 (12.4 %)	43 (10.8 %)	0.85 (0.61–1.17)	0.259	35 (7.8 %)	0.59 (0.41–0.84)	1.90E-03

The level of enrichment of the top 1,000 loci in each brain region was determined by Fisher’s exact test. 5hmC, 5-hydroxymethylcytosine; CI, confidence interval; A3SS, alternative 3’ splice site; A5SS, alternative 5’ splice site; AFE, alternative first exon; ALE, alternative last exon; CE, cassette exon; CNE, constitutive exon; EI, exon isoforms; II, intron isoforms; IR, intron retention; MXE, mutually exclusive exon

**Table 3:** The 1,000 top tissue-specific hydroxymethylated positions (TS-HMPs) between the prefrontal cortex and cerebellum are enriched in distinct genomic regions

	Loci with detectable 5hmC in brain (%)	Top 1,000 loci with tissue-specific differences		
		Sites with detectable 5hmC (%)	Enrichment (95 % CI)	*P* value
All probes (N = 79,263)		1,000	-	-
CpG island feature				
Island	7,837 (9.9 %)	317 (31.7 %)	4.23 (3.68–4.85)	6.37E-79
Shore	22,593 (28.5 %)	209 (20.9 %)	0.66 (0.57–0.77)	6.20E-08
Shelf	11,674 (14.7 %)	92 (9.2 %)	0.59 (0.47–0.73)	2.64E-07
Outside	36,342 (45.8 %)	381 (38.1 %)	0.73 (0.64–0.83)	9.82E-07
Unannotated	817 (1.0 %)	1 (0.1 %)	0.10 (0.00–0.54)	7.00E-04
Gene feature				
Intergenic	9,604 (12.1 %)	132 (13.2 %)	1.10 (0.91–1.33)	0.306
Distal promoter	3,703 (4.7 %)	25 (2.5 %)	0.52 (0.34–0.78)	6.30E-04
Proximal promoter	16,979 (21.4 %)	121 (12.1 %)	0.50 (0.41–0.61)	3.32E-14
Gene body	46,238 (58.3 %)	690 (69.0 %)	1.59 (1.39–1.82)	5.15E-12
Downstream	1,922 (2.4 %)	31 (3.1 %)	1.29 (0.87–1.85)	0.178
Unannotated	817 (1.0 %)	1 (0.1 %)	0.10 (0.00–0.54)	7.00E-04
Transcription factor binding site	25,482 (32.1 %)	125 (12.5 %)	0.30 (0.25–0.36)	1.30E-46
Dnase 1 hypersensitivity site	8,155 (10.3 %)	280 (28.0 %)	3.39 (2.94–3.91)	3.51E-54
Alternative transcription events (N = 30,659)		404	-	-
A3SS	798 (2.6 %)	14 (3.5 %)	1.34 (0.72–2.29)	0.270
A5SS	827 (2.7 %)	17 (4.2 %)	1.58 (0.91–2.59)	0.087
AFE	9,159 (29.9 %)	92 (22.8 %)	0.69 (0.54–0.88)	1.77E-03
ALE	3,017 (9.8 %)	40 (9.9 %)	1.01 (0.71–1.40)	0.933
CE	16,604 (54.2 %)	202 (50.0 %)	0.85 (0.69–1.04)	0.097
CNE	3,755 (12.2 %)	69 (17.1 %)	1.48 (1.12–1.92)	4.70E-03
EI	50 (0.2 %)	1 (0.2 %)	1.52 (0.04–8.91)	0.487
II	6,003 (19.6 %)	59 (14.6 %)	0.70 (0.52–0.93)	0.011
IR	3,529 (11.5 %)	54 (13.4 %)	1.19 (0.87–1.59)	0.240
MXE	3,814 (12.4 %)	39 (9.7 %)	0.75 (0.53–1.05)	0.095

The level of enrichment of the top 1,000 loci was determined by Fisher’s exact test. 5hmC, 5-hydroxymethylcytosine; CI, confidence interval; A3SS, alternative 3’ splice site; A5SS, alternative 5’ splice site; AFE, alternative first exon; ALE, alternative last exon; CE, cassette exon; CNE, constitutive exon; EI, exon isoforms; II, intron isoforms; IR, intron retention; MXE, mutually exclusive exon

### Some sites are characterized by considerable inter-individual variation in 5hmC

Given the hypothesized role of 5hmC in health and disease, we were interested in identifying regions of the genome characterized by inter-individual variation in 5hmC within both the prefrontal cortex (Additional file 2: Table S9) and cerebellum (Additional file 2: Table S10). The 1,000 top-ranked variable 5hmC sites were enriched in CpG islands both in the prefrontal cortex: (OR = 1.72, *P* = 4.03E-09) and the cerebellum (OR = 3.92, *P* = 2.41E-69) (Table 4; Additional file 1: Figure S3A). Furthermore, in both tissues, highly-variable 5hmC sites were under-represented in intergenic regions (Additional file 1: Figure S3B) (prefrontal cortex: OR = 0.84, *P* = 0.032; cerebellum: OR = 0.63, *P* = 3.86E-05) and in the proximal promoter (prefrontal cortex: OR = 0.76, *P* = 1.10E-03; cerebellum: OR = 0.47, *P* = 1.03E-16). However, there were some differences between the genic location of the most variable sites between the prefrontal cortex and the cerebellum; the most variable sites in the prefrontal cortex were under-represented in CpG island shelves (OR = 0.69, *P* = 1.82E-07), while the most variable sites in the cerebellum were under-represented in CpG island shores (OR = 0.52, *P* = 9.25E-17). Furthermore, in the gene body there was a significant over-representation of the most variable sites in the cerebellum (OR = 1.28, *P* = 1.78E-04), but not in the prefrontal cortex (OR = 1.00, *P* = 1.000). Interestingly over-/under-representation of sites at alternative events (Additional file 1: Figure S3C) was only seen for the most variable probes in the cerebellum and not in the prefrontal cortex, indicating that 5hmC may play a role in regulating gene expression through splicing in a tissue-specific manner. Despite observing genic differences in the most variable sites between individuals in the prefrontal cortex and cerebellum, there was a significant correlation in the inter-individual differences between regions, with the 1,000 most variable sites in the prefrontal cortex showing a similar degree of variability in the cerebellum (Additional file 1: Figure S4A; R = 0.30, *P* = 6.14E-17), and similarly the most variable sites in the cerebellum showing a similar degree of variability in the prefrontal cortex (Additional file 1: Figure S4B; R = 0.24, *P* = 5.4E-12).

**Table 4:** The most variable hydroxymethylated loci in prefrontal cortex and cerebellum are enriched in distinct genomic regions

	Loci with detectable 5hmC in brain (%)	Top 1,000 most variable loci in prefrontal cortex			Top 1,000 most variable loci in cerebellum		
		Sites with detectable 5hmC (%)	Enrichment (95 % CI)	*P* value	Sites with detectable 5hmC (%)	Enrichment (95 % CI)	*P* value
All probes (N = 79,263)		1,000	-	-	1,000	-	-
CpG island feature							
Island	7,837 (9.9 %)	159 (15.9 %)	1.72 (1.44–2.05)	4.037E-09	301 (30.1 %)	3.92 (3.41–4.51)	2.41E-69
Shore	22,593 (28.5 %)	270 (27.0 %)	0.93 (0.80–1.07)	0.307	171 (17.1 %)	0.52 (0.44–0.61)	9.25E-17
Shelf	11,674 (14.7 %)	107 (10.7 %)	0.69 (0.56–0.85)	2.23E-04	134 (13.4 %)	0.90 (0.74–1.08)	0.261
Outside	36,342 (45.8 %)	376 (37.6 %)	0.71 (0.62–0.81)	1.82E-07	281 (28.1 %)	0.46 (0.40–0.53)	3.04E-30
Unannotated	817 (1.0 %)	88 (8.8 %)	9.26 (7.28–11.67)	4.97E-50	113 (11.3 %)	12.23 (9.85–15.09)	1.10E-74
Gene feature							
Intergenic	9,604 (12.1 %)	99 (9.9 %)	0.80 (0.64–0.98)	0.032	80 (8.0 %)	0.63 (0.49–0.79)	3.86E-05
Distal promoter	3,703 (4.7 %)	36 (3.6 %)	0.76 (0.53–1.06)	0.113	30 (3.0 %)	0.63 (0.42–0.91)	0.010
Proximal promoter	16,979 (21.4 %)	172 (17.2 %)	0.76 (0.64–0.90)	1.10E-03	113 (11.3 %)	0.47 (0.38–0.57)	1.03E-16
Gene body	46,238 (58.3 %)	583 (58.3 %)	1.00 (0.88–1.14)	1.000	642 (64.2 %)	1.28 (1.12–1.46)	1.78E-04
Downstream	1,922 (2.4 %)	22 (2.2 %)	0.91 (0.56–1.38)	0.756	22 (22.0 %)	0.91 (0.56–1.38)	0.756
Unannotated	817 (1.0 %)	88 (8.8 %)	9.26 (7.28–11.67)	4.97E-50	113 (11.3 %)	12.23 (9.85–15.09)	1.10E-74
Transcription factor binding site	25,482 (32.1 %)	298 (29.8 %)	0.90 (0.78–1.03)	0.117	242 (24.2 %)	0.67 (0.58–0.78)	4.57E-08
Dnase 1 hypersensitivity site	8,155 (10.3 %)	77 (7.7 %)	0.73 (0.57–0.92)	6.33E-03	61 (6.1 %)	0.57 (0.43–0.74)	4.56E-06
Alternative transcription events (N = 30,659)		385	-	-	418	-	-
A3SS	798 (2.6 %)	15 (3.9 %)	1.52 (0.84–2.55)	0.144	15 (3.6 %)	1.39 (0.77–2.34)	0.214
A5SS	827 (2.7 %)	10 (2.6 %)	0.96 (0.46–1.80)	1.000	17 (4.1 %)	1.53 (0.88–2.49)	0.094
AFE	9,159 (29.9 %)	103 (26.8 %)	0.86 (0.68–1.08)	0.197	82 (19.6 %)	0.57 (0.44–0.73)	2.55E-06
ALE	3,017 (9.8 %)	36 (9.4 %)	0.95 (0.65–1.34)	0.863	38 (9.1 %)	0.92 (0.64–1.28)	0.679
CE	16,604 (54.2 %)	200 (51.9 %)	0.92 (0.74–1.13)	0.410	187 (44.7 %)	0.69 (0.56–0.84)	1.38E-04
CNE	3,755 (12.2 %)	60 (15.6 %)	1.32 (0.98–1.75)	0.051	86 (20.6 %)	1.86 (1.44–2.37)	1.95E-06
EI	50 (0.2 %)	1 (0.3 %)	1.59 (0.04–9.35)	0.471	2 (0.5 %)	2.94 (0.35–11.27	0.155
II	6,003 (19.6 %)	74 (19.2 %)	0.98 (0.75–1.27)	0.897	69 (16.5 %)	0.81 (0.62–1.06)	0.121
IR	3,529 (11.5 %)	41 (10.6 %)	0.92 (0.64–1.27)	0.688	68 (16.3 %)	1.49 (1.13–1.95)	4.24E-03
MXE	3,814 (12.4 %)	40 (10.4 %)	0.82 (0.57–1.14)	0.244	31 (7.4 %)	0.56 (0.38–0.82)	1.26E-03

The level of enrichment of the top 1,000 loci in each brain region was determined by Fisher’s exact test. 5hmC, 5-hydroxymethylcytosine; CI, confidence interval; A3SS, alternative 3’ splice site; A5SS, alternative 5’ splice site; AFE, alternative first exon; ALE, alternative last exon; CE, cassette exon; CNE, constitutive exon; EI, exon isoforms; II, intron isoforms; IR, intron retention; MXE, mutually exclusive exon

### A small proportion of DMPs identified by epigenome-wide association studies (EWAS) using standard BS approaches may actually reflect differences in 5hmC

**Fig. 5:**
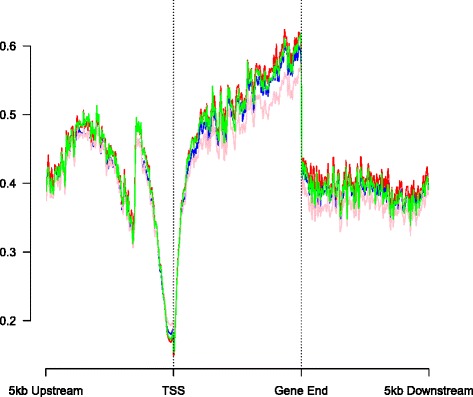
Canonical differences in DNA methylation in standard BS-treated DNA can be masked by 5hmC differences. There was no difference in levels of DNA modifications (5mC + 5hmC) in BS-treated DNA between the prefrontal cortex and the cerebellum. However, when examining true 5mC levels (oxBS) there are higher levels of DNA methylation at the 3’ end of the gene body in the prefrontal cortex than the cerebellum. Key: BS-treated (5mC + 5hmC levels) prefrontal cortex DNA (red), BS-treated (5mC + 5hmC levels) cerebellum DNA (green), oxBS-treated (5mC levels only) prefrontal cortex DNA (blue), oxBS-treated (5mC level only) prefrontal cortex DNA (pink)

Standard BS-treatment has been used in conjunction with the Illumina 450K methylation array in a growing number of EWAS analyses to identify differences in DNA methylation associated with exposure and disease. Given that this approach actually provides a cumulative measure of both 5mC and 5hmC, it is plausible that variation in 5hmC could confound findings that have largely been attributed to variation in DNA methylation. When we identified regions with the greatest difference in BS-generated data between the prefrontal cortex and cerebellum (Additional file 2: Table S11), these changes significantly correlated with differences in oxBS-generated data (R = 0.537, *P* = 6.85E-37) (Additional file 1: Figure S5; Additional file 2: Table S11). However, differences at some loci appeared to be driven by 5hmC, rather than 5mC variation, with eight of the top 1,000 BS tissue differences being driven more by a 5hmC difference than a 5mC difference. Looking across a canonical gene, we actually see no difference in DNA modification levels between the prefrontal cortex and the cerebellum when using standard BS-treated DNA (Fig. 5); however, we do see a considerable difference in true DNA methylation levels as determined using oxBS-treated DNA, with higher levels at the 3’ end of the gene in the prefrontal cortex than the cerebellum. We therefore recommend running BS and oxBS 450K arrays in parallel for investigating the role of DNA methylation in cross-tissue studies of complex disease.

## Conclusions

This study demonstrates the utility of combining oxBS-treatment with the Illumina 450k methylation array to systematically quantify 5hmC across the genome. Our study highlights region-specific patterns of 5hmC in the human brain, with overall higher levels observed in the cerebellum than the prefrontal cortex, and notable differences in the genomic location of the most hydroxymethylated loci between these brain regions. Loci demonstrating the greatest differences between brain regions (TS-HMPs) are highly enriched at CpG islands and in the gene body. We also identify considerable inter-individual variation in 5hmC at a subset of loci within each brain region, with these variable regions being particularly enriched in CpG islands and depleted in intergenic regions and the proximal promoter. Finally, we were able to confirm our findings in a second independent set of matched prefrontal cortex and cerebellum samples. Given the enrichment of 5hmC in the vicinity of genes involved in nervous system development and function, and the inability to distinguish this modification from 5mC using standard BS-based methods, we propose that approaches described here can be used to interrogate the role of 5hmC in neurological/neuropsychiatric phenotypes and disease.

## Methods

### Sample preparation

Our discovery cohort comprised prefrontal cortex and cerebellum samples dissected from eight individuals. First, brain tissue from six control donors, with no evidence of neurological impairment, was obtained from the London Neurodegenerative Disease Brain Bank (LNDBB) (http://www.kcl.ac.uk/ioppn/depts/bcn/Our-research/Neurodegeneration/brain-bank.aspx). For each sample, genomic DNA was extracted from 100 mg of tissue using a standard phenol-chloroform extraction method. Additionally, two control cerebellum samples, provided by CEGX, were run alongside. Sample characteristics for all discovery samples are detailed in Additional file 2: Table S12. Validation data were generated from matched prefrontal cortex and cerebellum samples from 18 control donors, with no evidence of neurological impairment, currently being profiled as part of an independent study in our lab.

### Bisulfite (BS) and oxidative-bisulfite (oxBS) treatment

For each sample DNA was treated using: (1) the Zymo BS conversion kit; (2) the Cambridge Epigenetix BS conversion module; and (3) the Cambridge Epigenetix oxBS conversion module. For Zymo BS, 500 ng DNA from each sample was sodium BS-treated using the Zymo EZ 96 DNA methylation kit (Zymo Research) according to the manufacturer’s standard protocol. For CEGX BS and CEGX oxBS samples, 4 μg of high molecular weight genomic DNA was sheared into <10 kb fragments using a Covaris g-Tube. The fragmented DNA was subsequently concentrated into a total volume of 40 μL by passing the sample through a geneJET purification column. The 40 μL was split into two 20 μL samples and processed using the TrueMethyl kit following the manufacturer’s instructions. We performed enzyme digestion of the CEGX conversion controls as recommended by the manufacturer, and all samples showed satisfactory conversion (see Additional file 1: Figure S6 for example output).

### Illumina Infinium BeadArray

DNA modifications were quantified using the Illumina Infinium Human 450K Methylation Array according to the manufacturer’s instructions, with minor amendments. In brief, the DNA input to the MSA4 plate for whole genome amplification was increased from 4 μL to 7 μL of CEGX BS/oxBS treated DNA. To compensate for the increased DNA volume, the concentration of NaOH was increased to 0.4 M and only 1 μL added to the MSA4 plate.

### Quality control (QC) and data normalization

All computations and statistical analyses were performed within the R statistical environment (version 3.1.2) [27] and Bioconductor [28]. Signal intensities were imported into R using the *methylumi* package [29]. Initial QC checks were performed to assess concordance between reported and predicted gender. Non-CpG SNP probes on the array were used to confirm that samples where sourced from the same individual were genetically identical (Additional file 1: Figure S7). Data were pre-processed using *wateRmelon* (version 1.4.0) [30], with a custom *P* filter threshold of 5 % of sites with a detection *P* value <0.05. No precedents have yet been set for pre-processing and normalizing oxBS data. We therefore tested all of the different normalization strategies available within the *wateRmelon* package. We found that although other normalization strategies scored highly within each metric, data analyzed using *dasen* consistently scored well for each metric (Additional file 2: Table S13), and were therefore used for data normalization. Non-CpG SNP probes, probes that have been reported to contain common (MAF >5 %) SNPs in the CG or single base extension position, or probes that were non-specific or mismapped [31, 32], were flagged and disregarded in the evaluation of our results, leaving 374,094 probes for analysis.

### Data analysis

The level of 5-hmC within each sample was identified by subtracting the oxBS (CEGX) beta-value from the BS (CEGX) beta value at each probe on the 450K array (Δβ_BS-oxBS_) in each sample. A threshold for detection of 5hmC was established by determining the lowest fifth percentile in the data (that is, -0.09158275 in this study). We then applied this value as a threshold for the positive data. Sites with an average 5hmC level in either the prefrontal cortex or cerebellum above this level (that is, +0.09158275) were classified as having ‘detectable’ levels of 5hmC. Illumina 450K array probes were annotated using ENCODE annotation [25], and Fisher’s exact test was used to determine if 5hmC was enriched in specific genomic regions. Normalized 5hmC levels are available on our online analytical database HABIT for the 79,263 sites we identified as having ‘detectable’ 5hmC in one or both brain regions (http://epigenetics.iop.kcl.ac.uk/HMC/).

### Pathway analyses

Illumina UCSC gene annotation was used to create a test gene list from the hydroxymethylated probes in the prefrontal cortex (N = 37,145) and the cerebellum (N = 65,563), or the TS-HMPs (N = 1,000). A logistic regression approach was used to test if gene lists predicted pathway membership while controlling for the number of probes annotated to each gene. Pathways were downloaded from the Gene Ontology website (http://geneontology.org/) and all genes annotated to parent terms were also included. All genes with at least one Illumina probe annotated and annotated to at least one GO pathway were considered. Pathways were filtered to those with between 10 and 2,000 genes in them. After applying this method to all pathways, significant pathways (*P* <0.05) were taken and grouped where overlapping genes explained the signal. This was achieved by taking the most significant pathway, and retesting all remaining significant pathways while controlling additionally for the best term. If the test genes no longer predicted the pathway, the term was said to be explained by the most significant pathway, and hence these pathways were grouped together. This algorithm was repeated, taking the next most significant term, until all pathways were considered as the most significant or found to be explained by a more significant term.

### Determining 5mC and 5hmC in a canonical gene

A sliding window approach was used to calculate average 5mC and 5hmC levels in a gene. To investigate canonical differences in 5hmC we calculated the moving average for 5mC (oxBS) and 5hmC (Δβ_BS-oxBS_) in the 79,263 loci deemed to be hydroxymethylated for overlapping 1 % sliding windows from 5 kb upstream of the gene to 5 kb downstream of the gene. To investigate canonical differences in data derived from BS-treated DNA, which may be driven by 5hmC differences we calculated the moving average for BS-treated DNA (5mC + 5hmC) and for oxBS DNA (5mC) in the 374,094 loci that passed QC for overlapping 1 % sliding windows from 5 kb upstream of the gene to 5 kb downstream of the gene.

### Ethics

Brain samples were obtained from the London Neurodegenerative Disease Brain Bank (LNDBB) (http://www.kcl.ac.uk/ioppn/depts/bcn/Our-research/Neurodegeneration/brain-bank.aspx). Samples were provided with informed consent according to the Declaration of Helsinki (1991) and ethical approval for the study was provided by the NHS South East London REC 3.

### Data availability

All microarray data have been uploaded to GEO and are available under accession number GSE62003.

## Additional files

Additional file 1:
**Supplementary Figures 1–7 in a single pdf, with legends embedded in each figure.** (PDF 1 mb) Additional file 2:
**Supplementary Tables 1–13 in a single Excel file as separate tabs, with legends embedded in each table.** (XLSX 14105 kb)

## Abbreviations

5caC: 5-carboxylcytosine
5fC: 5-formylcytosine
5hmC: 5-hydroxymethylcytosine
5mC: 5-methylcytosine
A3SS: Alternative 3’ splice site
A5SS: Alternative 5’ splice site
AFE: Alternative first exon
ALE: Alterntive last exon
BS: Bisulfite treatment
CE: Cassette exon
CGI: CpG island
CNE: Constitutive exon
EI: Exon isoforms
EWAS: Epigenome-wide association study
II: Inron isoforms
IR: Intron retention
MXE: Mutually exclusive exon
oxBS: Oxidative-bisulfite treatment
QC: Quality control
