# Erratum to: Variation in 5-hydroxymethylcytosine across human cortex and cerebellum

**DOI:** 10.1186/s13059-016-0958-4

**Published:** 2016-06-17

**Authors:** Katie Lunnon, Eilis Hannon, Rebecca G.Smith, Emma Dempster, Chloe Wong, Joe Burrage, Claire Troakes, Safa Al-Sarraj, Agnieszka Kepa, Leonard Schalkwyk, Jonathan Mill

**Affiliations:** University of Exeter Medical School, RILD, University of Exeter, Barrack road, Devon, UK; Institute of Psychiatry, Psychology and Neuroscience, King’s College London, De Crespigny Park, London, UK; University of Essex, Wivenhoe Park, Colchester, CO4 3SQ UK

After the publication of this work [1] it was noticed that the GEO accession number in the data availability section was incorrect. This has now been corrected in the original version of this paper.
