# Supplementary material for: Variation in 5-hydroxymethylcytosine across human cortex and cerebellum
Source: Genome Biol. 2016 Feb 16;17:27. doi: 10.1186/s13059-016-0871-x (PMC4756397; doi:10.1186/s13059-016-0871-x)
Supplement: Additional file 1: — Supplementary Figures 1–7 in a single pdf, with legends embedded in each figure. (PDF 1 mb) [file 13059_2016_871_MOESM1_ESM.pdf]

Supplementary Figure 1: Comparison of different bisulfite treatment methods. (**A**) Following data normalization with the Dasen function in the R package WateRmelon we compared the mean betas between Zymo BS, CEGX BS and CEGX oxBS. The average beta value in Zymo BS was highly correlated with CEGX BS in prefrontal cortex (**B**) (R^2=0.985, P<2.2E-16) and (**C**) cerebellum (R^2=0.991, P<2.2E-16).

A

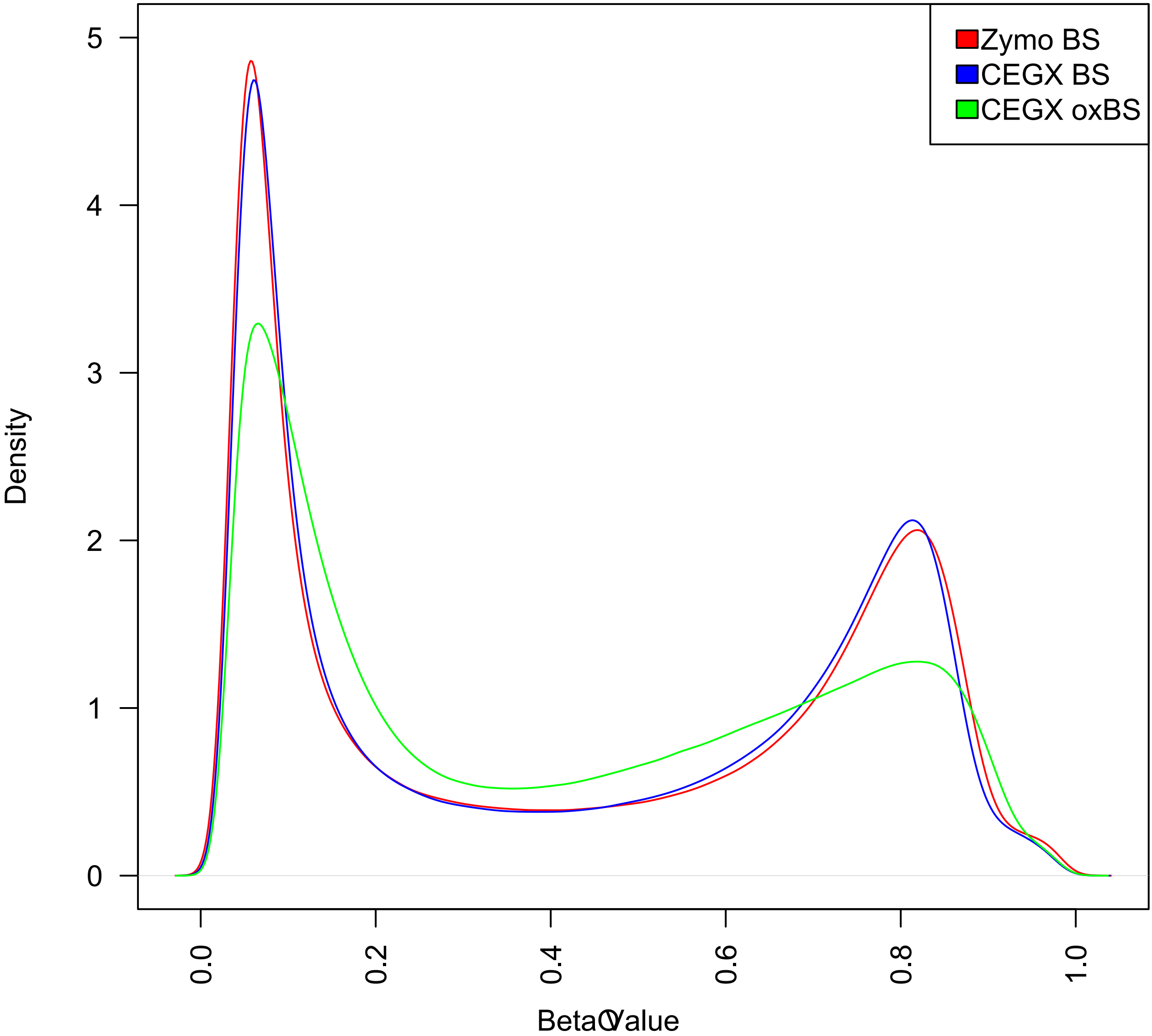

Zymo BS
CEGX BS
CEGX oxBS
Density
Beta Value
0
1
2
3
4
5
0.0
0.2
0.4
0.6
0.8
1.0

B

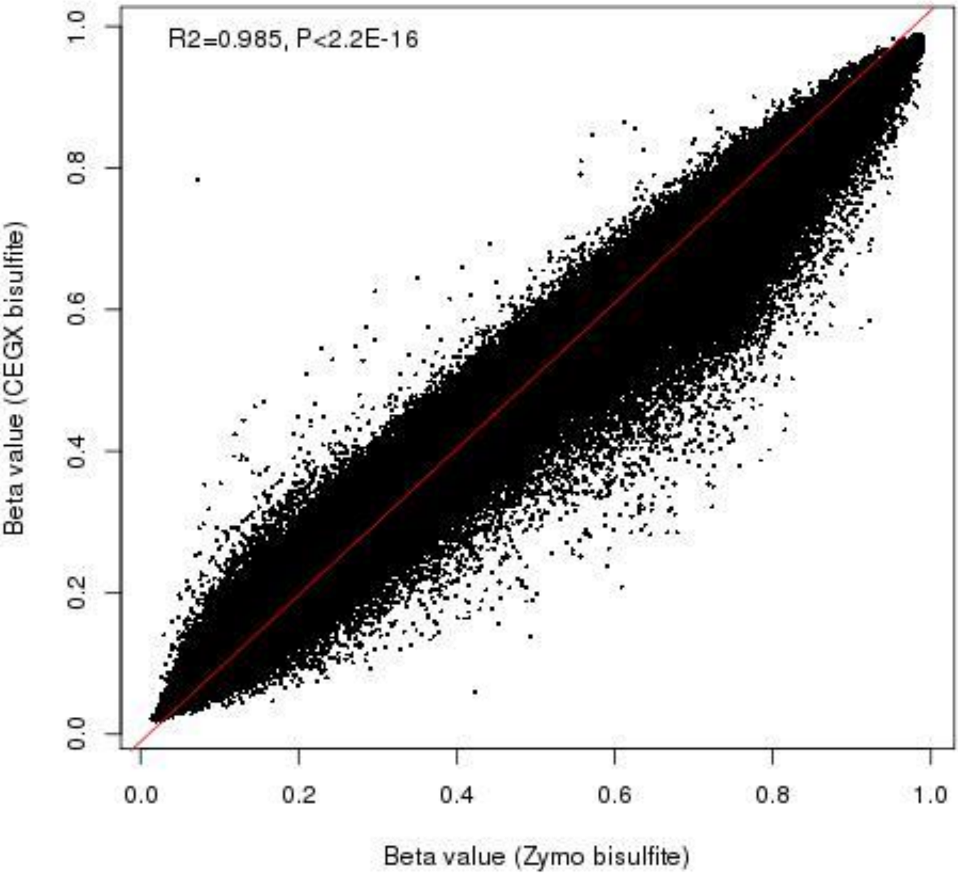
R2=0.985, P<2.2E-16
Beta value (CEGX bisulfite)
Beta value (Zymo bisulfite)
0.0
0.2
0.4
0.6
0.8
1.0

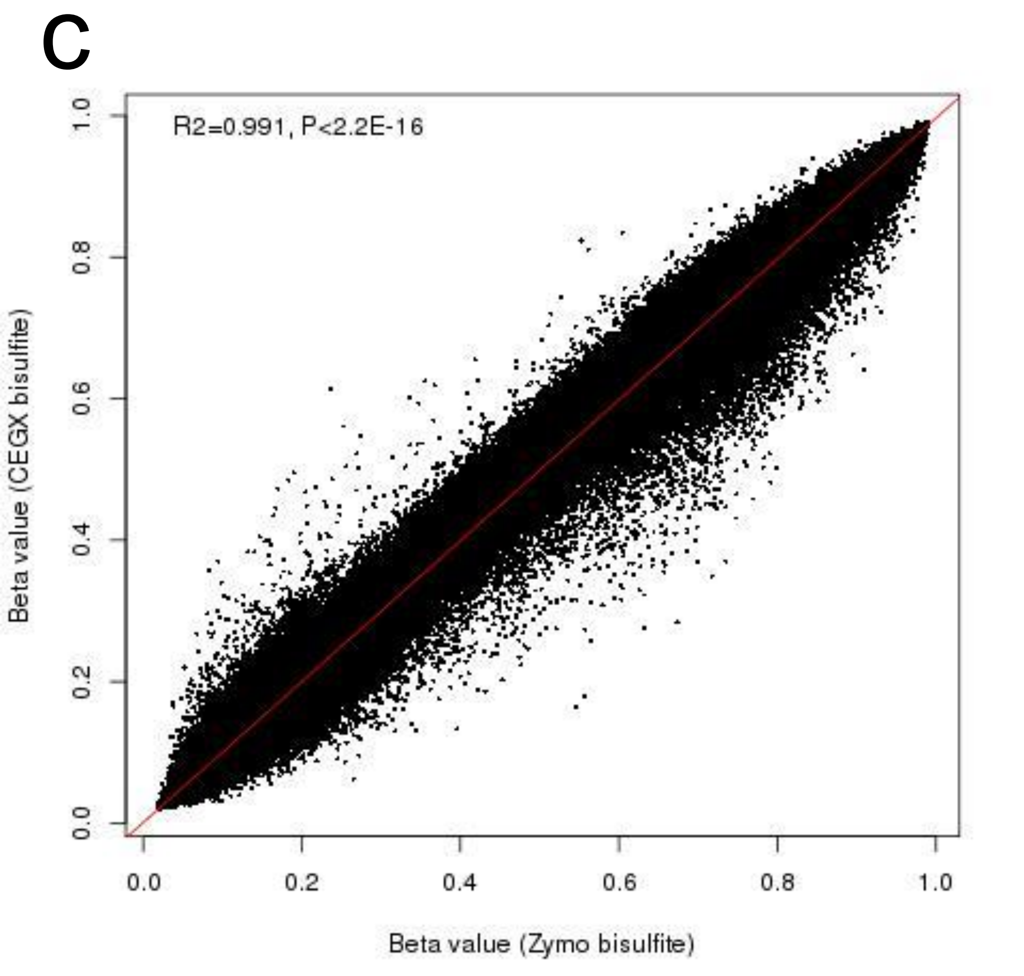
C
R2=0.991, P<2.2E-16
Beta value (CEGX bisulfite)
Beta value (Zymo bisulfite)
0.0
0.2
0.4
0.6
0.8
1.0

Supplementary Figure 2: The top TS-HMPs showed distinct differences in 5hmC level between the prefrontal cortex and the cerebellum. Shown are the top six TS-HMPs as ranked by % 5hmC difference; (**A**) cg25439798, (**B**) cg08446824, (**C**) cg04781796, (**D**) cg20182983, (**E**) cg04850731 and (**F**) cg16627786. The red dashed line denotes our threshold for "detectable" 5hmC of 0.09158275.

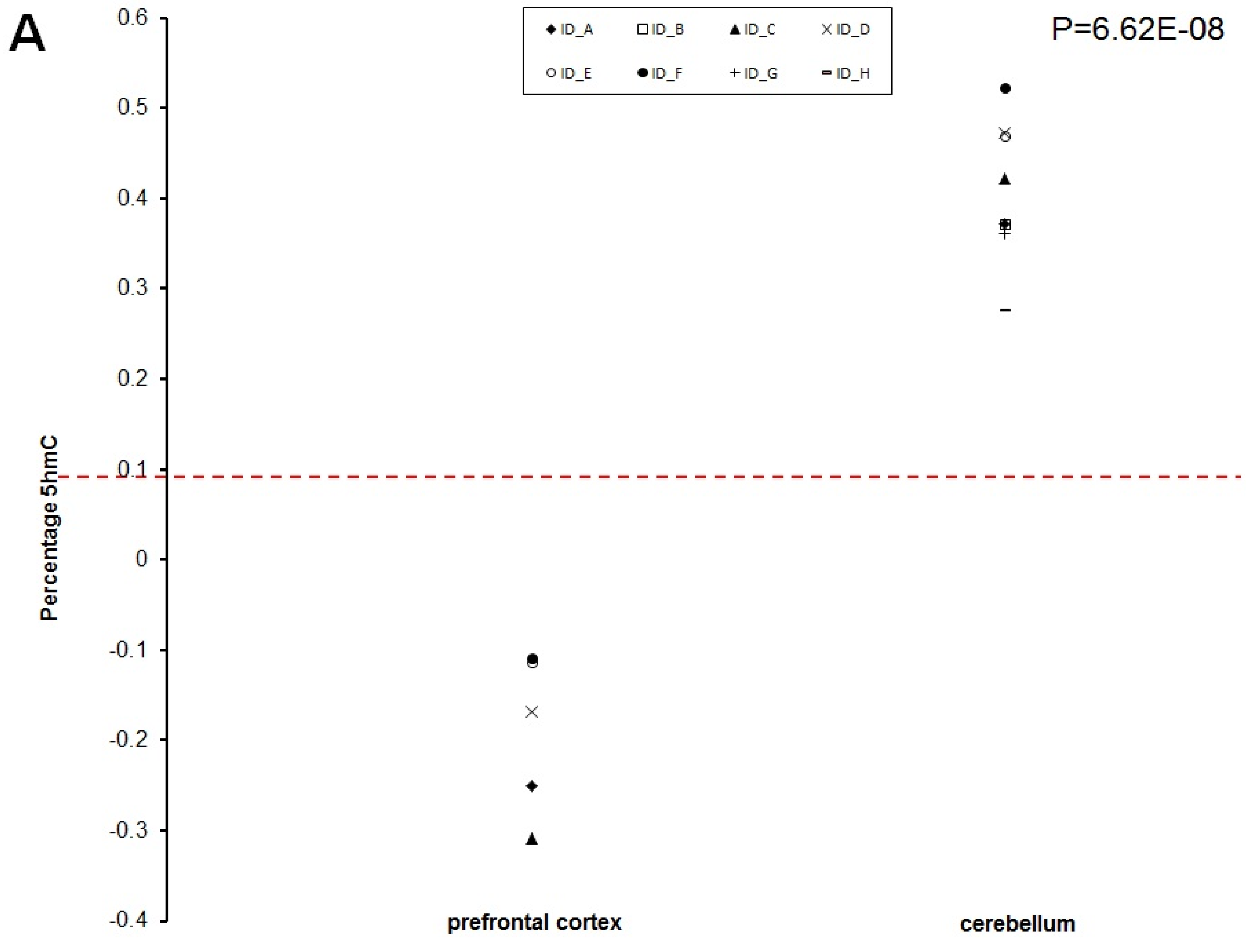
A
P=6.62E-08
ID_A
ID_B
ID_C
ID_D
ID_E
ID_F
ID_G
ID_H
Percentage 5hmC
0.6
0.5
0.4
0.3
0.2
0.1
0
-0.1
-0.2
-0.3
-0.4
prefrontal cortex
cerebellum

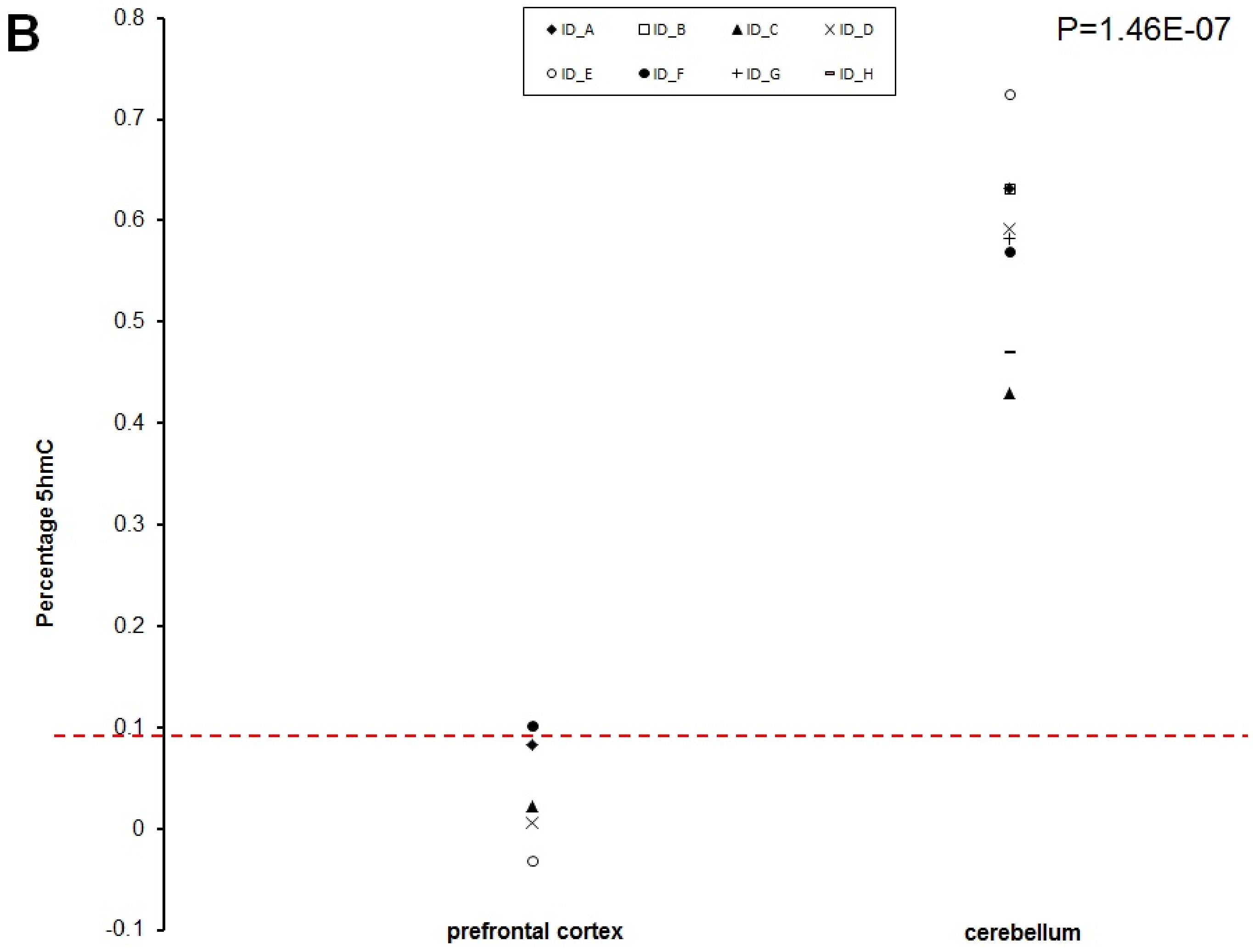
B
P=1.46E-07
ID_A
ID_B
ID_C
ID_D
ID_E
ID_F
ID_G
ID_H
Percentage 5hmC
0.8
0.7
0.6
0.5
0.4
0.3
0.2
0.1
0
-0.1
prefrontal cortex
cerebellum

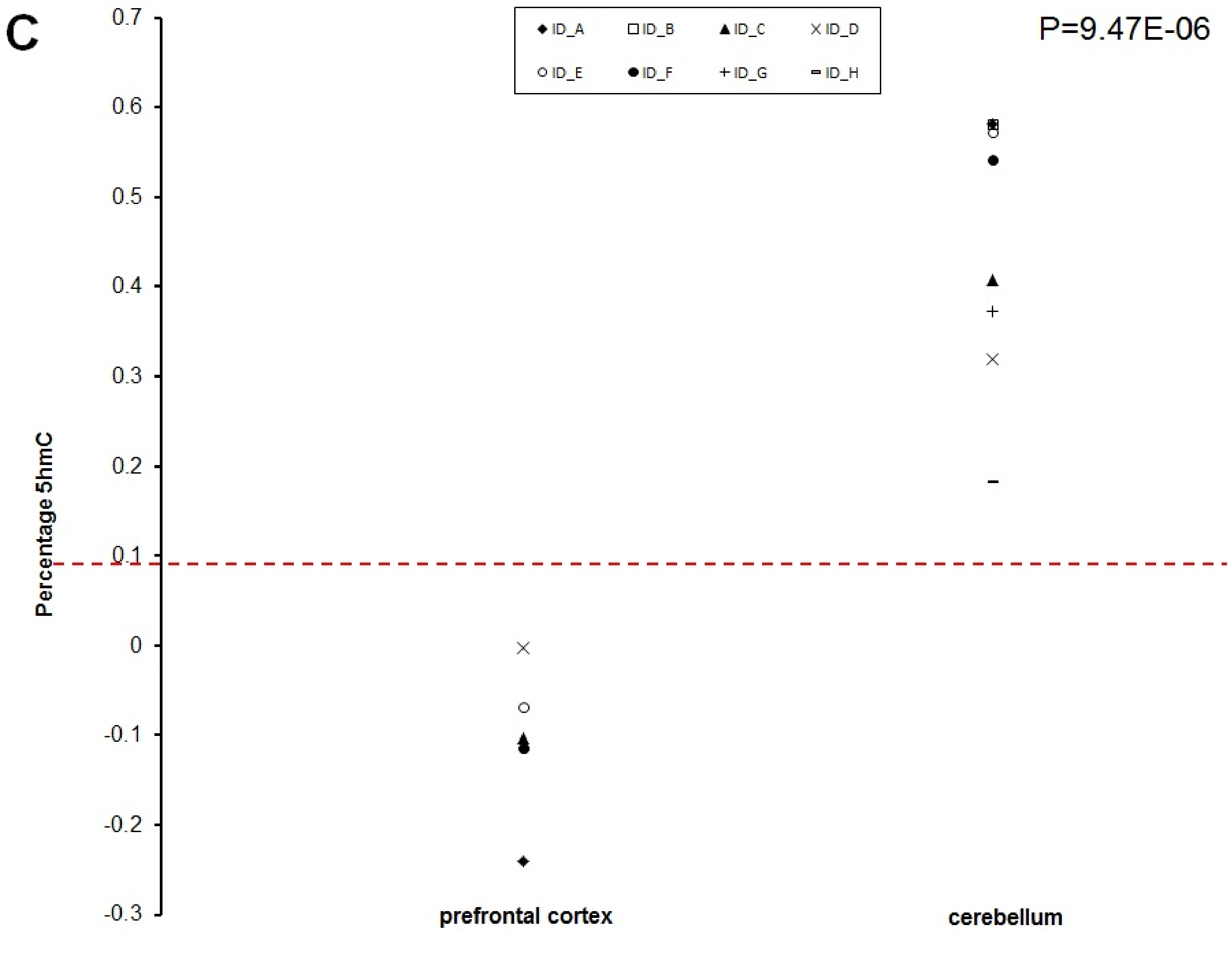
C
P=9.47E-06
ID_A
ID_B
ID_C
ID_D
ID_E
ID_F
ID_G
ID_H
0.7
0.6
0.5
0.4
0.3
0.2
0.1
0
-0.1
-0.2
-0.3
Percentage 5hmC
prefrontal cortex
cerebellum

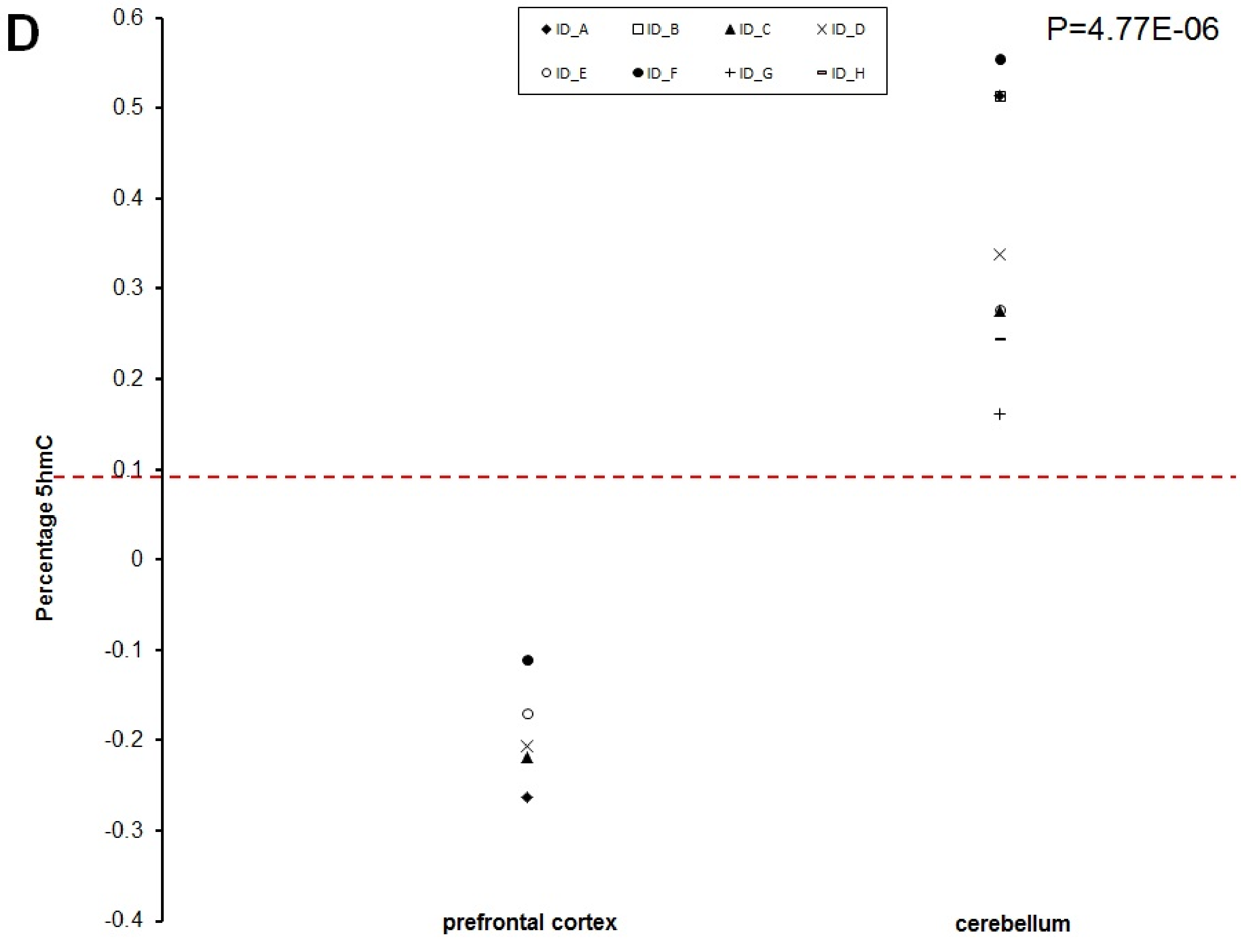
D
P=4.77E-06
ID_A
ID_B
ID_C
ID_D
ID_E
ID_F
ID_G
ID_H
Percentage 5hmC
0.6
0.5
0.4
0.3
0.2
0.1
0
-0.1
-0.2
-0.3
-0.4
prefrontal cortex
cerebellum

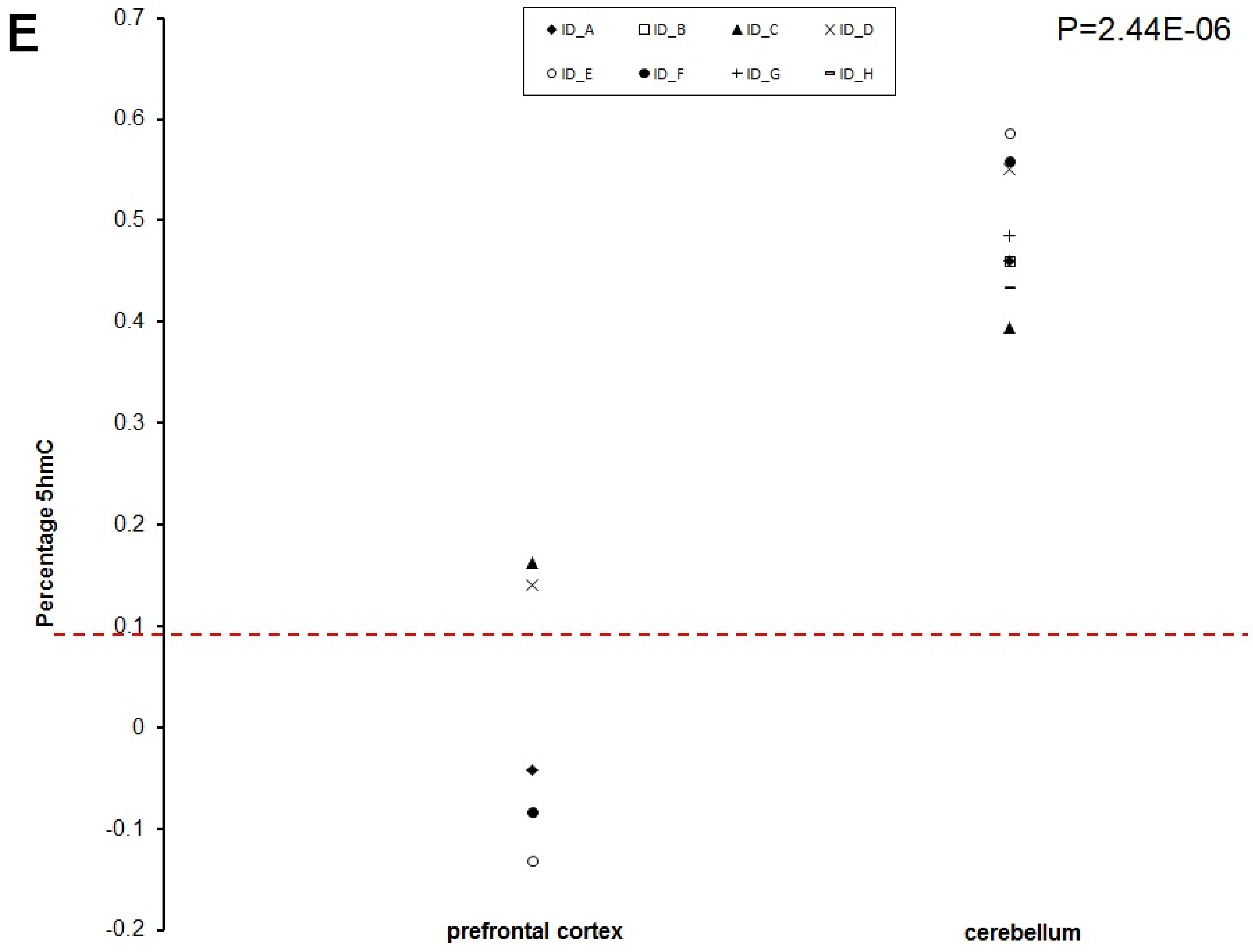
E
P=2.44E-06
ID_A
ID_B
ID_C
ID_D
ID_E
ID_F
ID_G
ID_H
Percentage 5hmC
0.7
0.6
0.5
0.4
0.3
0.2
0.1
0
-0.1
-0.2
prefrontal cortex
cerebellum

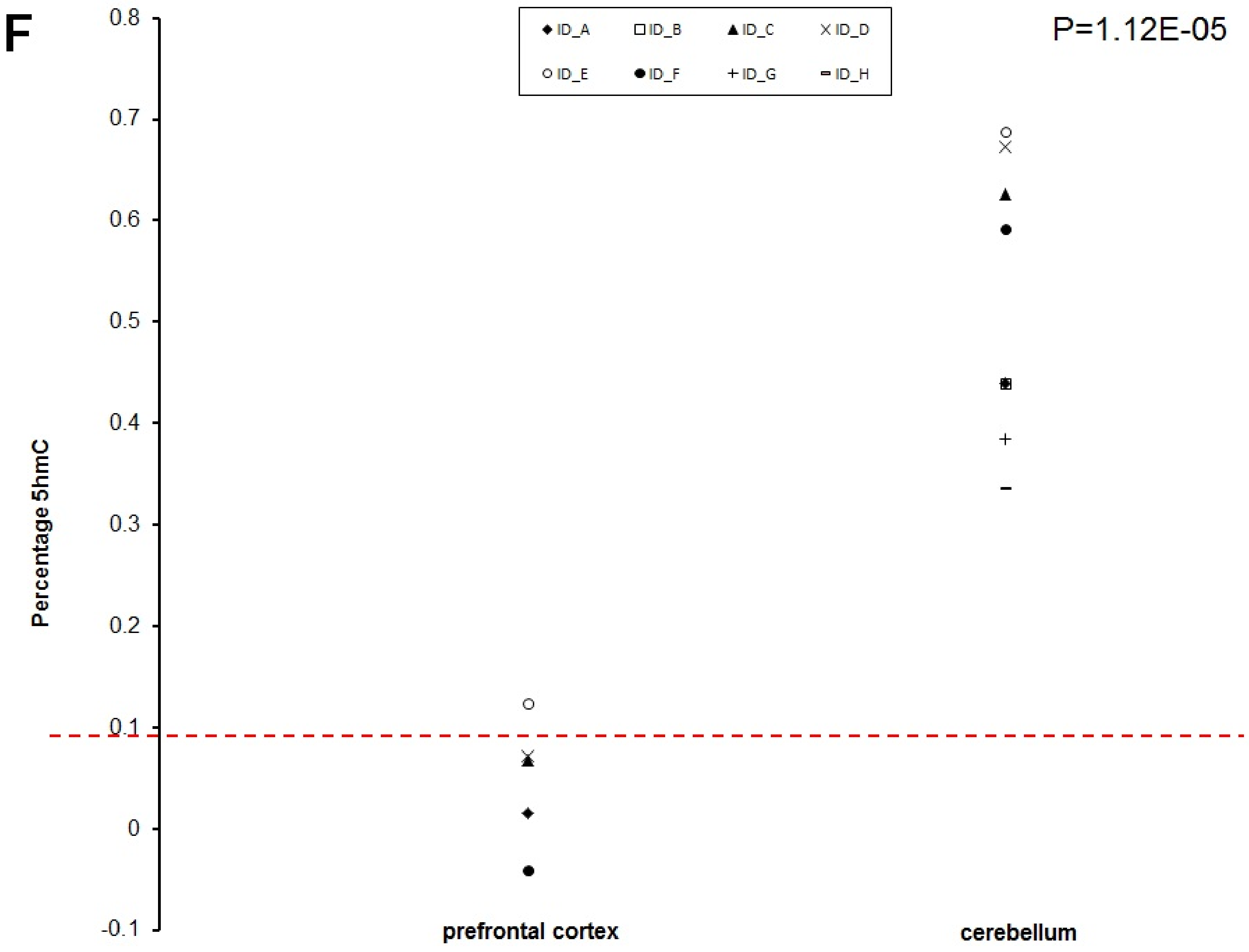

F
P=1.12E-05
ID_A
ID_B
ID_C
ID_D
ID_E
ID_F
ID_G
ID_H
Percentage 5hmC
0.8
0.7
0.6
0.5
0.4
0.3
0.2
0.1
0
-0.1
prefrontal cortex
cerebellum

Supplementary Figure 3: Sites characterized by the highest levels of 5hmC, tissue-specific differences in 5hmC, and inter-individual variation in 5hmC are seen in distinct genomic locations. The genomic distribution of these top 1,000 sites varied in their proximity to (**A**) CpG islands, (**B**) genomic position and (**C**) at alternative events, compared to the 79,263 loci with “detectable” 5hmC. Abbreviations: 3’ splice site (A3SS), alternative 5’ splice site (A5SS), alternative first exon (AFE), alternative last exon (ALE), cassette exon (CE); constitutive exon (CNE), exon isoforms (EI), intron isoforms (II), intron retention (IR), mutually exclusive exon (MXE).

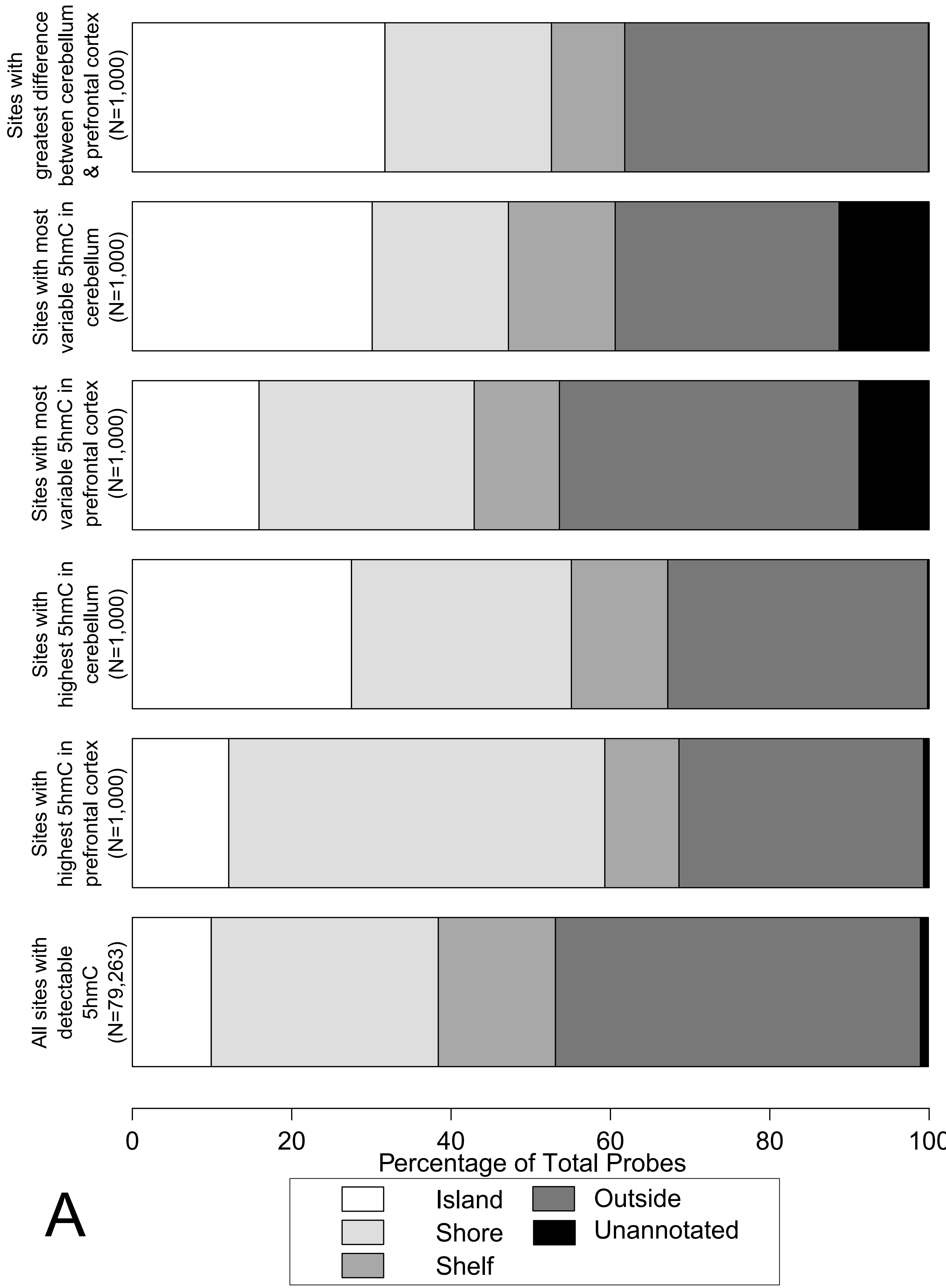
Sites with greatest difference between cerebellum & prefrontal cortex (N=1,000)
Sites with most variable 5hmC in cerebellum (N=1,000)
Sites with most variable 5hmC in prefrontal cortex (N=1,000)
Sites with highest 5hmC in cerebellum (N=1,000)
Sites with highest 5hmC in prefrontal cortex (N=1,000)
All sites with detectable 5hmC (N=79,263)
0
20
40
60
80
100
Percentage of Total Probes
Island
Shore
Shelf
Outside
Unannotated
A

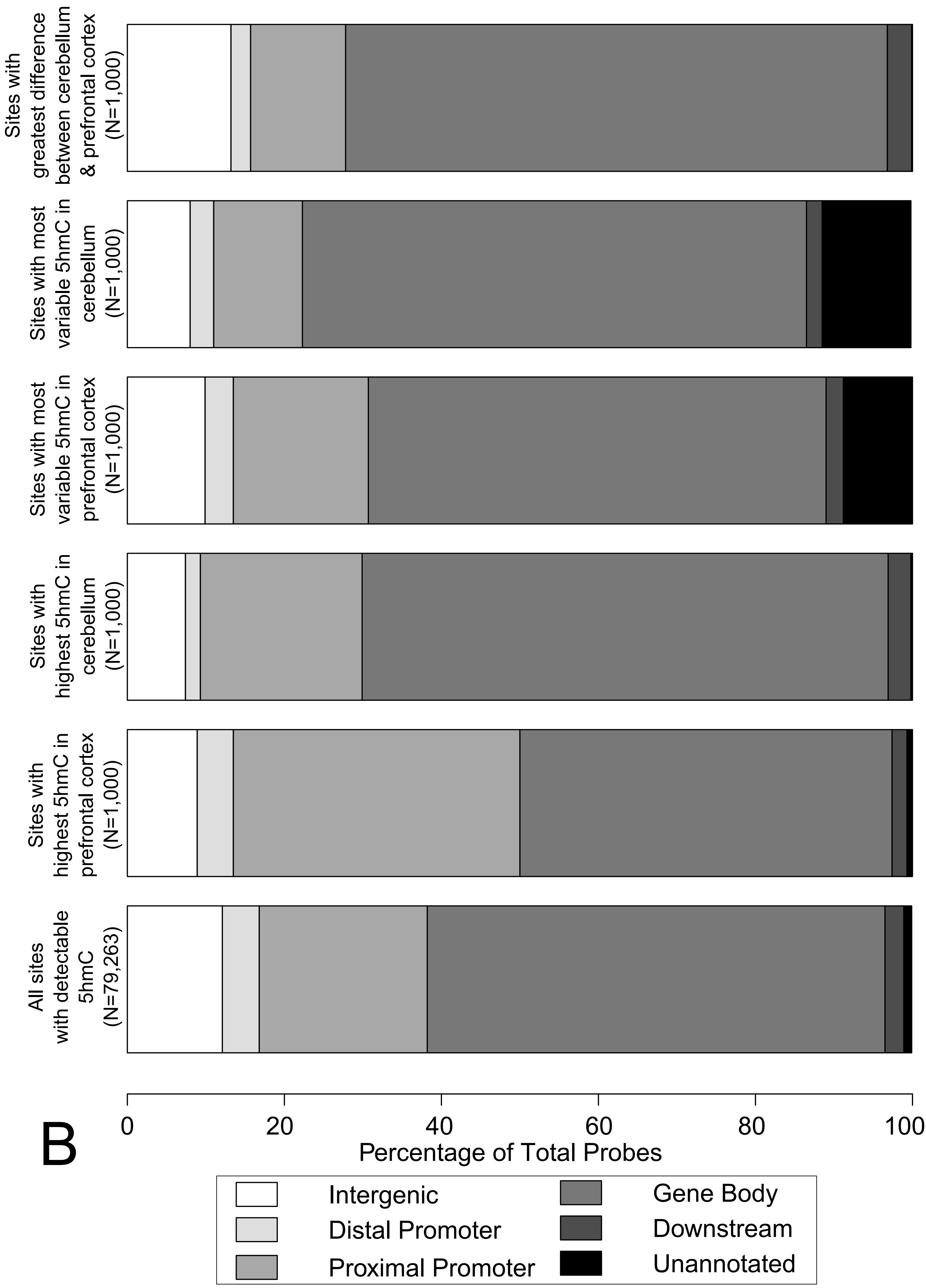
Sites with greatest difference between cerebellum & prefrontal cortex (N=1,000)
Sites with most variable 5hmC in cerebellum (N=1,000)
Sites with most variable 5hmC in prefrontal cortex (N=1,000)
Sites with highest 5hmC in cerebellum (N=1,000)
Sites with highest 5hmC in prefrontal cortex (N=1,000)
All sites with detectable 5hmC (N=79,263)
0
20
40
60
80
100
Percentage of Total Probes
B
Intergenic
Distal Promoter
Proximal Promoter
Gene Body
Downstream
Unannotated

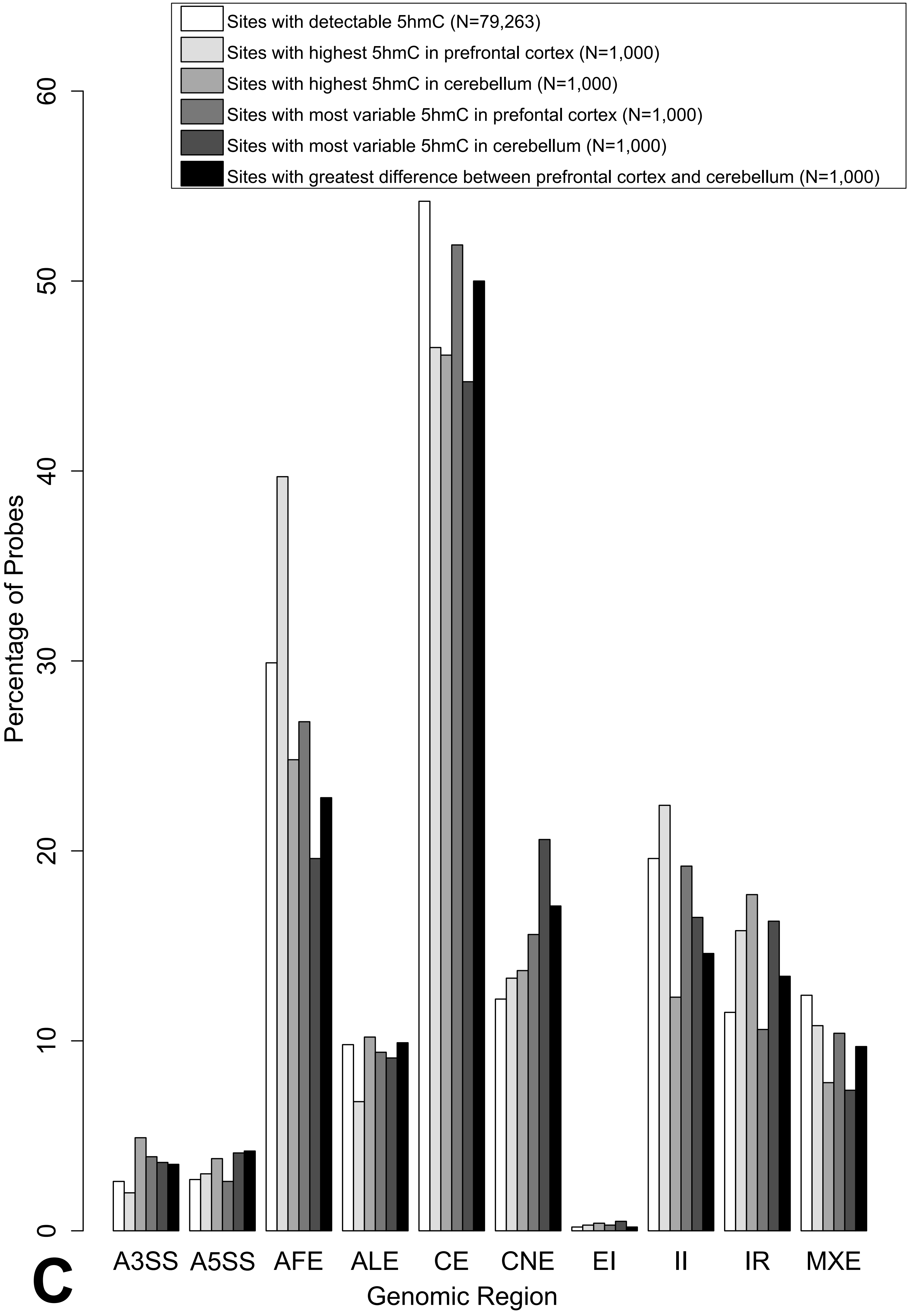
Sites with detectable 5hmC (N=79,263)
Sites with highest 5hmC in prefrontal cortex (N=1,000)
Sites with highest 5hmC in cerebellum (N=1,000)
Sites with most variable 5hmC in prefontal cortex (N=1,000)
Sites with most variable 5hmC in cerebellum (N=1,000)
Sites with greatest difference between prefrontal cortex and cerebellum (N=1,000)
Percentage of Probes
0
10
20
30
40
50
60
A3SS
A5SS
AFE
ALE
CE
CNE
EI
II
IR
MXE
Genomic Region
C

Supplementary Figure 4: Sites characterized by the greatest inter-individual variation in 5hmC are correlated between brain regions. The 1,000 probes with the greatest variation (standard deviation) between individuals in the prefrontal cortex were correlated with the variation in the same probes in the cerebellum (R=0.30, P=6.14E-17) (**A**). Similarly the 1,000 probes with the greatest variation between individuals in the cerebellum were correlated with the variation in the same probes in the prefrontal cortex (R=0.24, P=5.4E-12) (**B**).

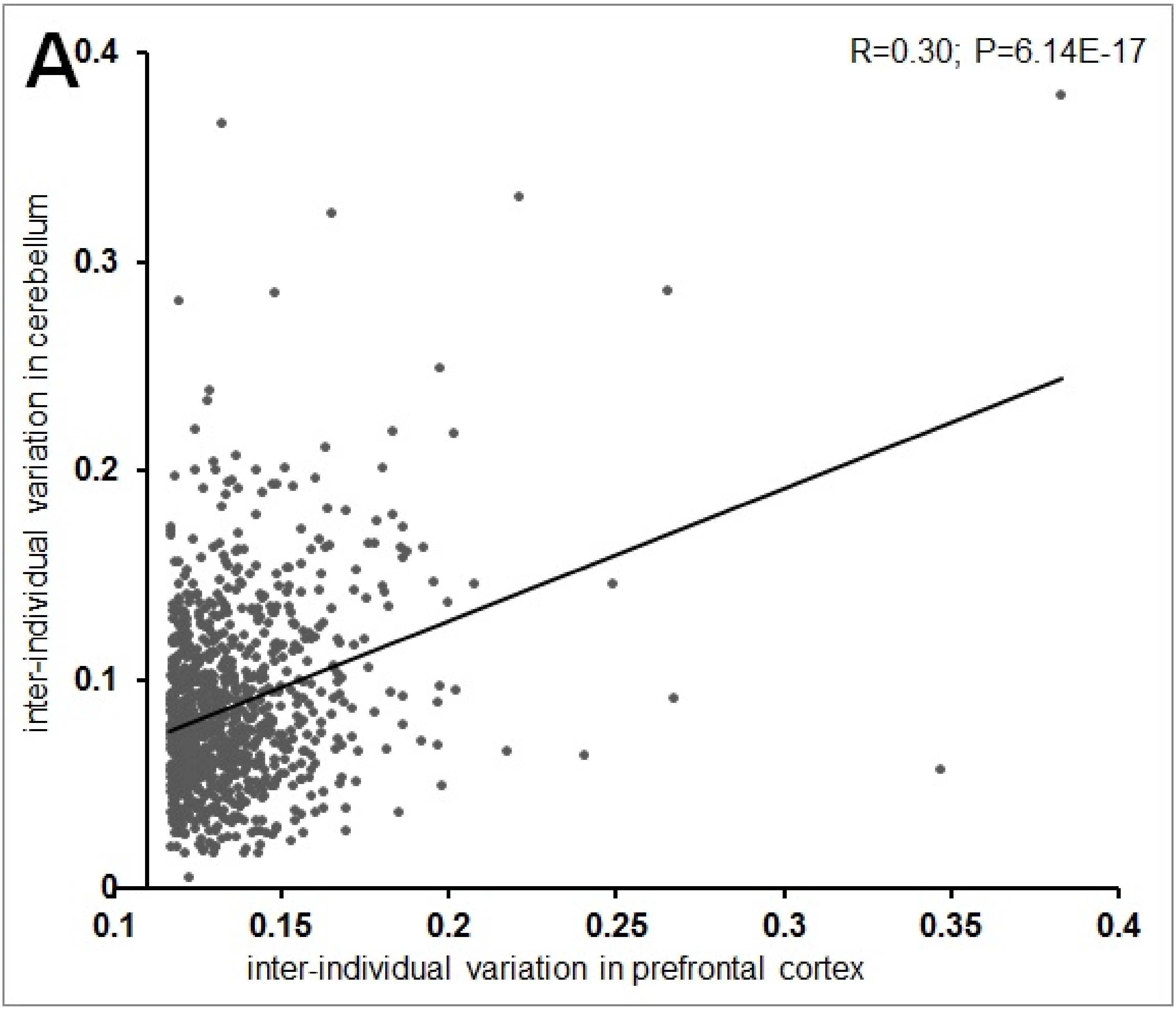

A
R=0.30; P=6.14E-17
0.4
0.3
0.2
0.1
0
0.1
0.15
0.2
0.25
0.3
0.35
0.4
inter-individual variation in cerebellum
inter-individual variation in prefrontal cortex

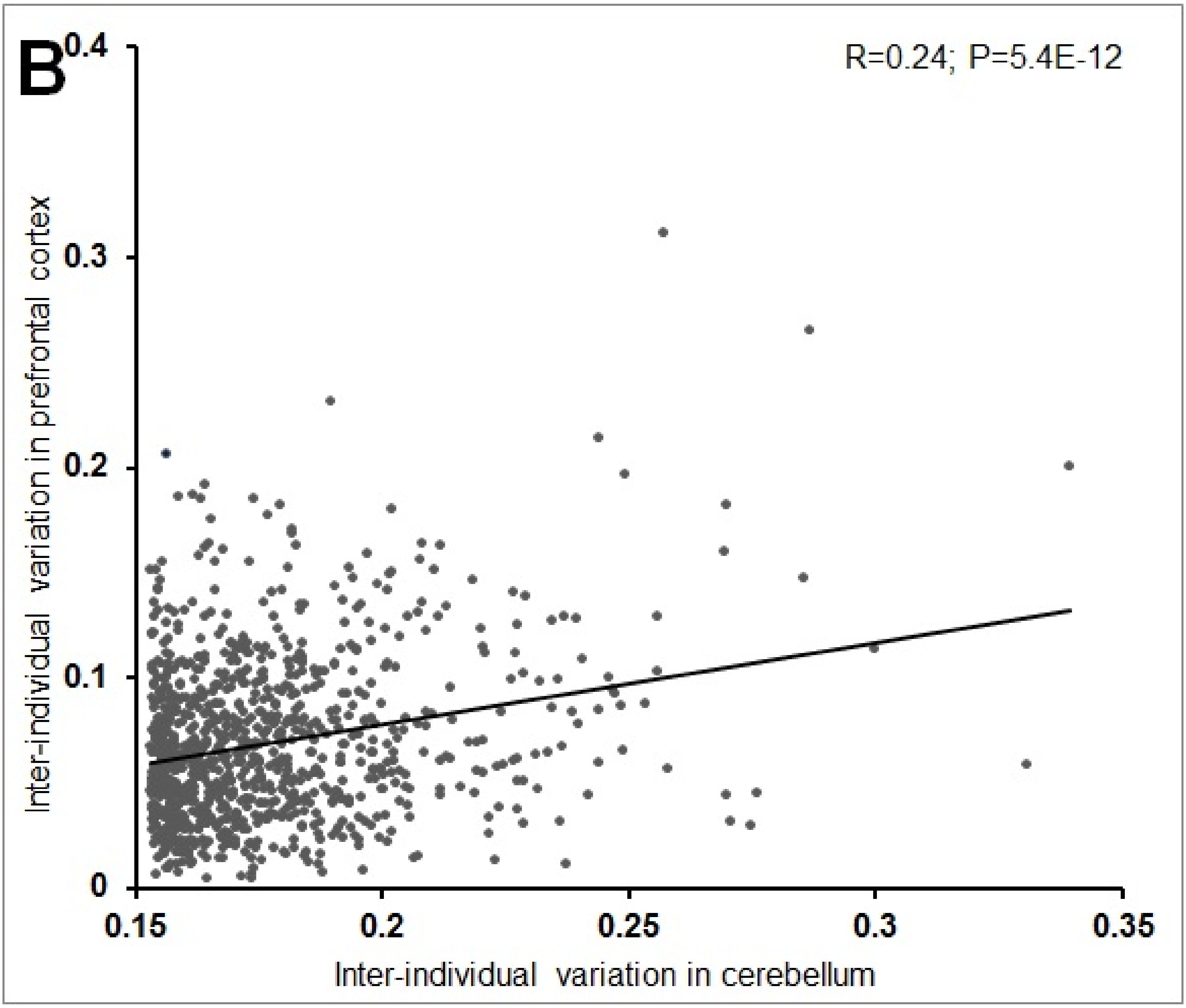
B
R=0.24; P=5.4E-12
Inter-individual variation in prefrontal cortex
0.4
0.3
0.2
0.1
0
0.15
0.2
0.25
0.3
0.35
Inter-individual variation in cerebellum

Supplementary Figure 5: Levels of DNA modifications in the top 1,000 loci with the greatest difference between brain regions in BS-generated data are highly correlated with brain region differences in oxBS-generated data. The 1,000 probes with the greatest difference between the prefrontal cortex and the cerebellum, as measured using BS-generated data (i.e. 5mC + 5hmC) are highly correlated with the difference between the prefrontal cortex and the cerebellum, as measured using oxBS-generated data (i.e. 5mC) (R=0.537, P=6.848E-37).

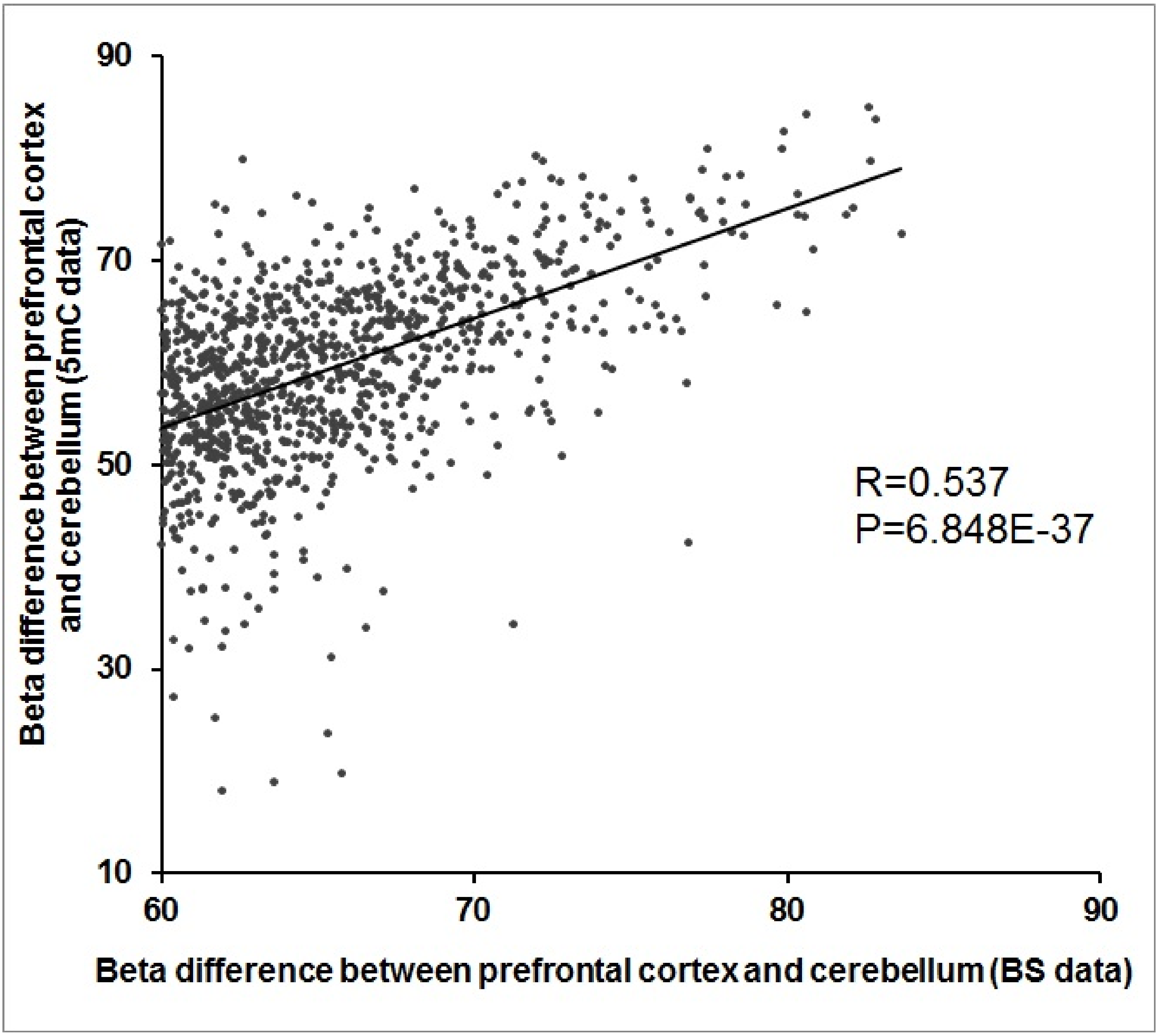

90
70
50
30
10
60
70
80
90
Beta difference between prefrontal cortex and cerebellum (5mC data)
Beta difference between prefrontal cortex and cerebellum (BS data)
R=0.537
P=6.848E-37

Supplementary Figure 6: CEGX conversion controls to demonstrate conversion of 5hmC. PCR amplification of oxBS (**A**) and BS (**B**) treated samples confirms a product for all samples. Following enzymatic digest the oxBS samples remain uncut, with the exception of the cutting control (**C**) while the BS samples are digested (**D**), demonstrating complete conversion of the 5hmC to 5fC.

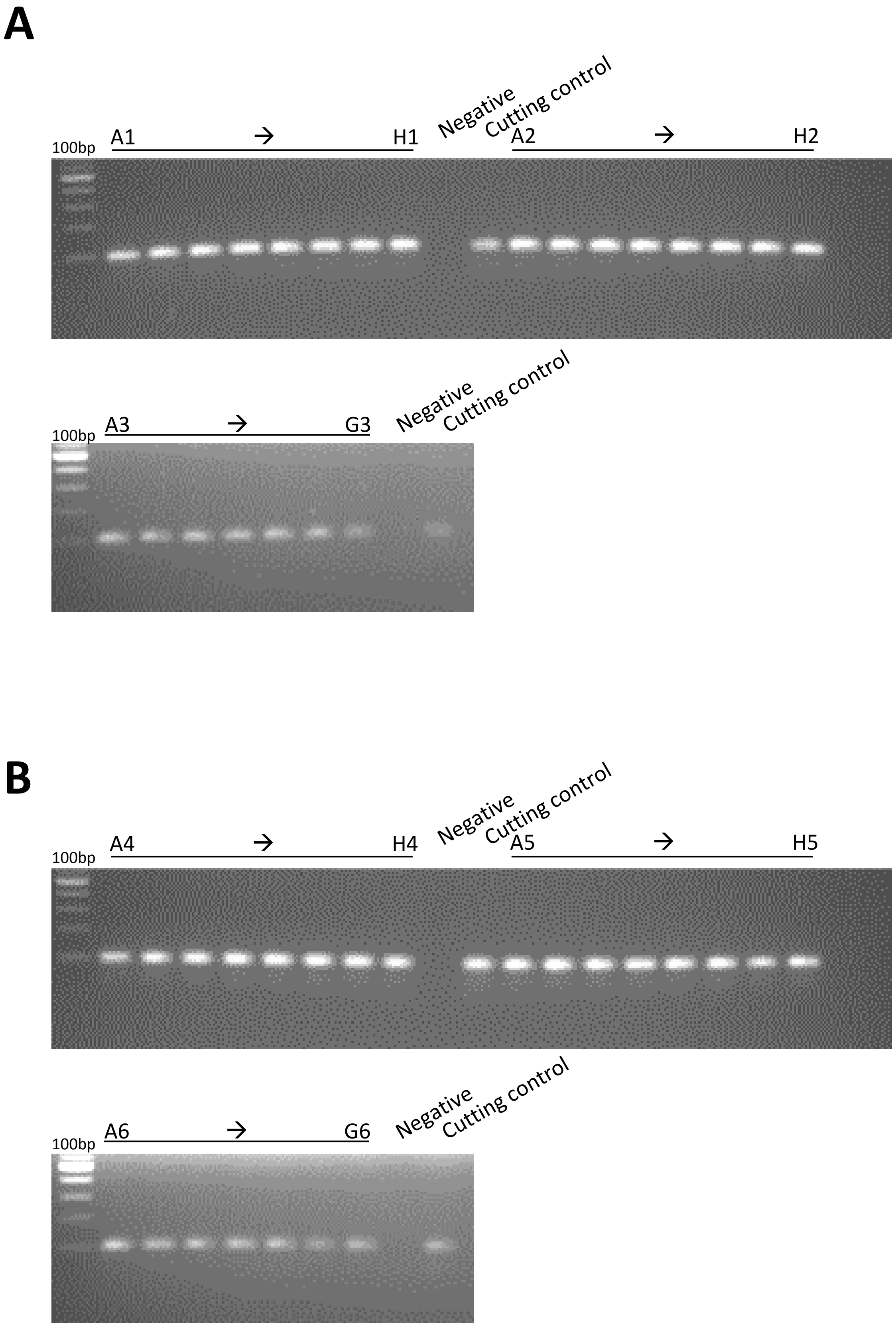
A
Negative
Cutting control
A1
H1
A2
H2
100bp
Negative
Cutting control
A3
G3
100bp
B
Negative
Cutting control
A4
H4
A5
H5
100bp
Negative
Cutting control
A6
G6
100bp

C

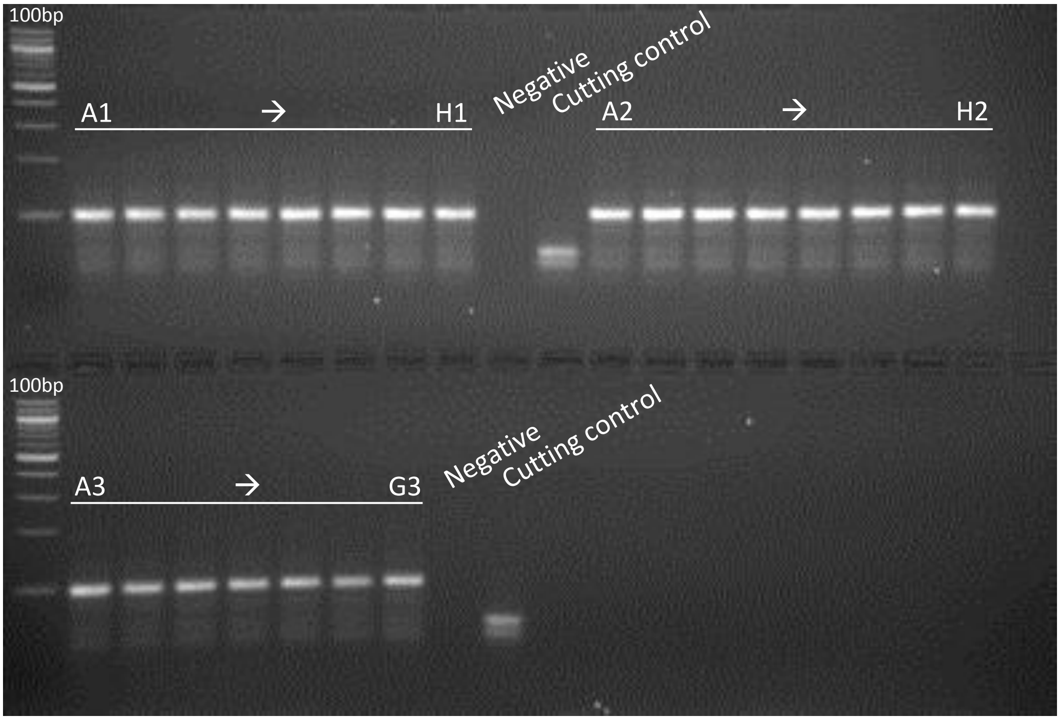
100bp
Negative
Cutting control
A1
H1
A2
H2
100bp
Negative
Cutting control
A3
G3

D

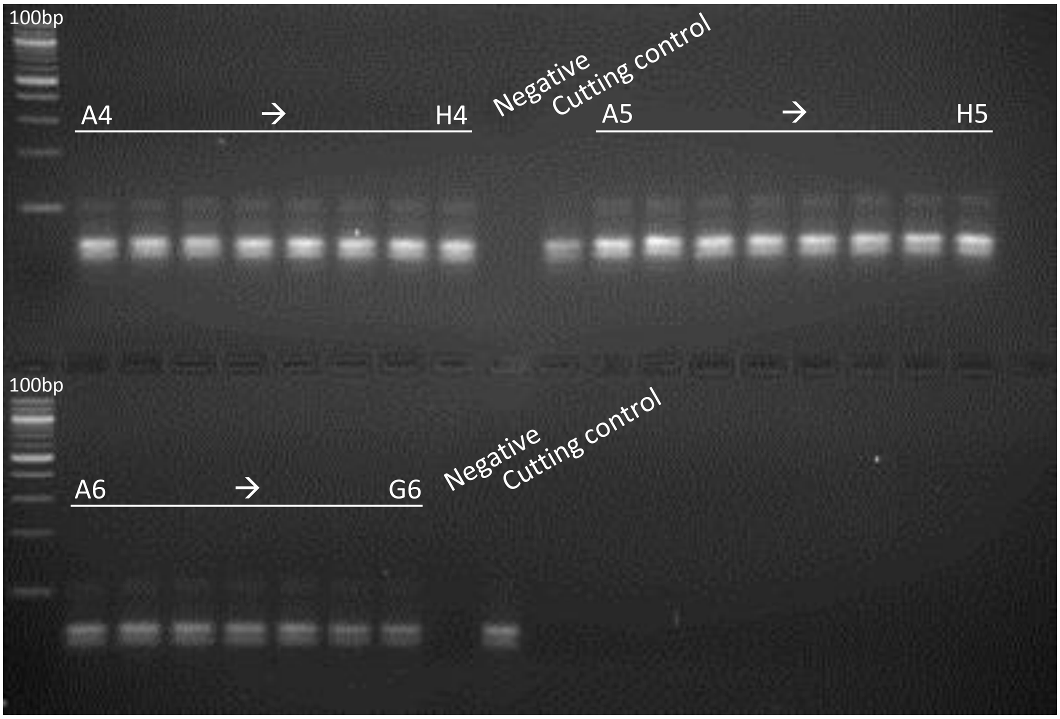
100bp
Negative
Cutting control
A4
H4
A5
H5
100bp
Negative
Cutting control
A6
G6

Supplementary Figure 7: Hierarchical cluster dendrogram using the 65 non-CpG SNP probes on the 450K array to demonstrate that matched prefrontal cortex and cerebellum samples were sourced from the same individuals.

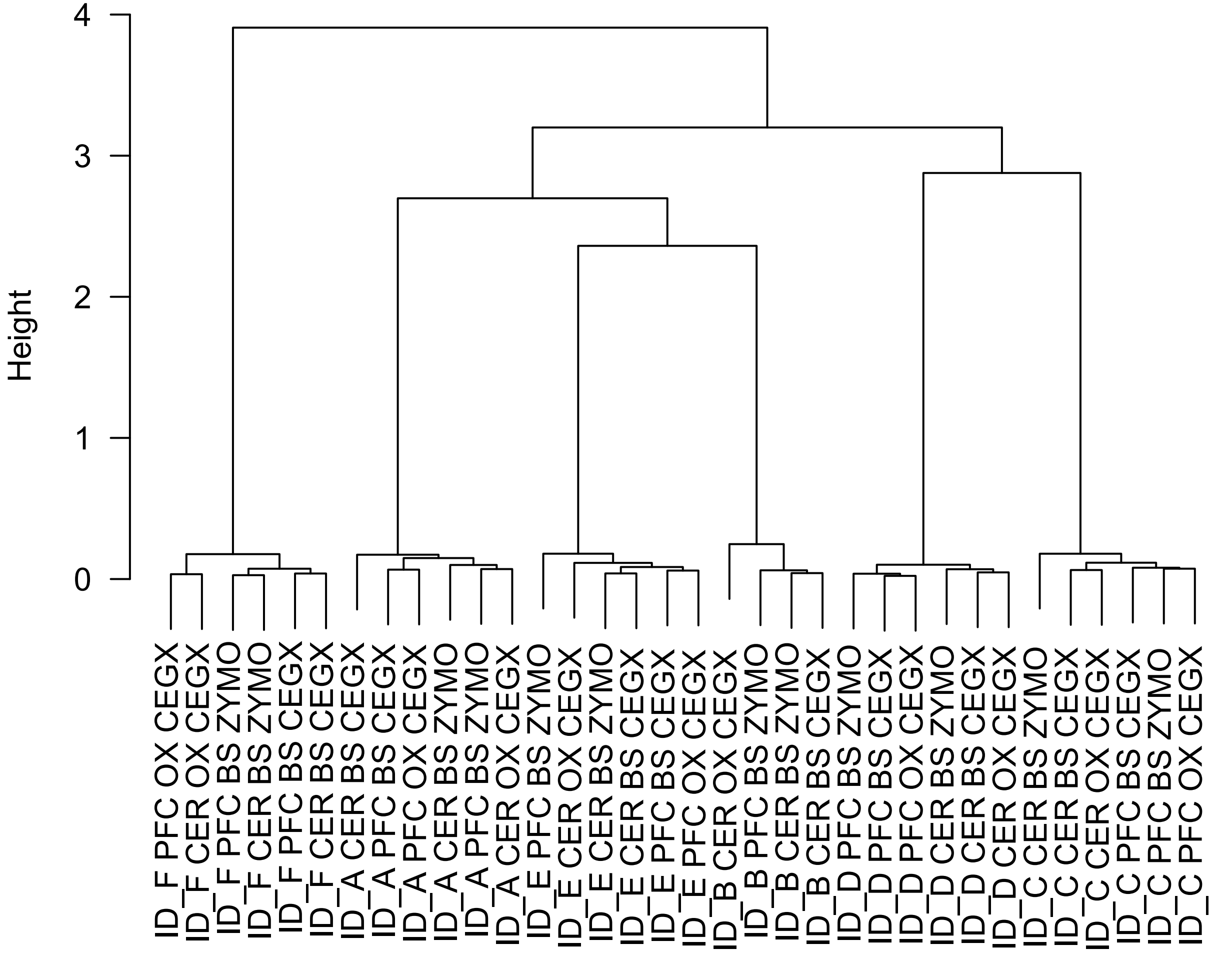
Height
0
1
2
3
4
ID_F PFC OX CEGX
ID_F CER OX CEGX
ID_F PFC BS ZYMO
ID_F CER BS ZYMO
ID_F PFC BS CEGX
ID_F CER BS CEGX
ID_A CER BS CEGX
ID_A PFC BS CEGX
ID_A PFC OX CEGX
ID_A CER BS ZYMO
ID_A PFC BS ZYMO
ID_A CER OX CEGX
ID_E PFC BS ZYMO
ID_E CER OX CEGX
ID_E CER BS ZYMO
ID_E CER BS CEGX
ID_E PFC BS CEGX
ID_E PFC OX CEGX
ID_B CER OX CEGX
ID_B PFC BS ZYMO
ID_B CER BS ZYMO
ID_B CER BS CEGX
ID_D PFC BS ZYMO
ID_D PFC BS CEGX
ID_D PFC OX CEGX
ID_D CER BS ZYMO
ID_D CER BS CEGX
ID_D CER OX CEGX
ID_C CER BS ZYMO
ID_C CER BS CEGX
ID_C CER OX CEGX
ID_C PFC BS CEGX
ID_C PFC BS ZYMO
ID_C PFC OX CEGX
